# The Potential of Technology-Based Psychological Interventions for Anorexia and Bulimia Nervosa: A Systematic Review and Recommendations for Future Research

**DOI:** 10.2196/jmir.3554

**Published:** 2015-03-31

**Authors:** Sandra Schlegl, Carolina Bürger, Luise Schmidt, Nirmal Herbst, Ulrich Voderholzer

**Affiliations:** ^1^Department of Psychiatry and PsychotherapyUniversity of MunichMünchenGermany; ^2^Department of Psychiatry and PsychotherapyFreiburgGermany; ^3^Schön Klinik RoseneckPrien am ChiemseeGermany

**Keywords:** anorexia nervosa, bulimia nervosa, computers, Internet, mobile phone, cognitive behavioral therapy

## Abstract

**Background:**

Previous studies have shown an unmet need in the treatment of eating disorders. In the last decade, interest in technology-based interventions (TBIs) (including computer- and Internet-based interventions [CBIs] or mobile interventions) for providing evidence-based therapies to individuals with different mental disorders has increased.

**Objective:**

The aim of this review was to systematically evaluate the potential of TBIs in the field of eating disorders, namely for anorexia nervosa (AN) and bulimia nervosa (BN), for both prevention and treatment, and also for carers of eating disorder patients.

**Methods:**

A systematic literature search was conducted using Medline and PsycINFO. Bibliographies of retrieved articles were also reviewed without date or study type restrictions.

**Results:**

Forty studies resulting in 45 publications reporting outcomes fulfilled the inclusion criteria: 22 randomized controlled trials, 2 controlled studies, and 16 uncontrolled studies. In total, 3646 patients were included. Overall, the studies provided evidence for the efficacy of guided CBIs, especially for BN patients and for compliant patients. Furthermore, videoconferencing also appeared to be a promising approach. Evaluation results of Internet-based prevention of eating disorders and Internet-based programs for carers of eating disorder patients were also encouraging. Finally, there was preliminary evidence for the efficacy of mobile interventions.

**Conclusions:**

TBIs may be an additional way of delivering evidence-based treatments to eating disorder patients and their use is likely to increase in the near future. TBIs may also be considered for the prevention of eating disorders and to support carers of eating disorder patients. Areas of future research and important issues such as guidance, therapeutic alliance, and dissemination are discussed.

## Introduction

Up to 4% of women have an eating disorder. Among these, the prevalence is 0.3% for anorexia nervosa (AN), 1% for bulimia nervosa (BN) [1], and 2.4% for eating disorders not otherwise specified (EDNOS) [2]. Evidence-based psychological treatments exist, especially for BN, and include cognitive behavioral therapy (CBT) and interpersonal psychotherapy (IPT) [3,4]. For adolescent AN patients, the efficacy of family-based therapy is apparent [3,4]. For adult AN patients, a combination of renourishment and psychotherapy (eg, CBT, supportive clinical management, IPT) is recommended [5]. There is still a lack of evidence-based treatments for adults with AN due to several methodological difficulties. These include difficulties in recruiting participants because this disorder is relatively rare, lack of treatment acceptance, and high dropout rates [6,7].

However, previous international studies on health service utilization showed an unmet need for the treatment of patients with eating disorders [8,9]. In their meta-analysis, Hart et al [10] found that only one-quarter of sufferers sought eating disorder-specific treatment. Barriers discussed were shame or fear of stigmatization when going to a psychologist. On the other hand, the number of patients seeking treatment in primary care has increased, especially for BN [11]. However, patients often undergo psychological treatment only after a considerable delay, and waiting times for treatment are long [12,13]. Furthermore, there is a general shortage of specialized therapists and institutions, and evidence-based methods are still lacking in routine care.

Technology-based interventions (TBIs), including computer- and Internet-based interventions (CBIs) or mobile interventions, have the potential to reach patients who otherwise may not access help, and to improve health care for those seeking treatment, by offering immediate access to evidence-based interventions. Communication in TBIs can take place synchronously (in real time, such as videoconference, chat rooms) or asynchronously (with lag between contacts, such as email, postings on a secure website, text messaging). Information exchange can occur in writing, just via audio communication, or via webcam. TBIs can be administered individually or in the form of group sessions.

There are various forms of CBIs that differ especially in their amount of therapist guidance:

1. Computer- and Internet-based unguided self-help (unguided CBI): this is the generic term for self-help interventions primarily delivered via computer technology. Available programs mainly are multimedia-based as well as CBT-based. Patients can either use them at home or in health care settings, and programs are designed to enable patients to work through them independent of a therapist.
2. Computer- and Internet-based guided self-help (guided CBI): support can range from screening for suitability, offering technical advice, monitoring progress and outcome, as well as giving emotional support [14].
3. Internet-based therapist-delivered treatments: these can be delivered using different methods, such as email, chat rooms, and videoconferencing, either solely Internet-delivered or in combination with face-to-face treatment. Patients have regular contact with a therapist.

In the last decade, a large body of empirical evidence on the acceptance and efficacy of CBIs for mental disorders has accrued. Several reviews and meta-analyses have shown that new CBI treatments hold great promise in the treatment of adults with depression [15-17], depression and anxiety [18,19], anxiety [20,21], obsessive-compulsive disorder [22,23], and traumatic stress [24,25]. CBIs have been found to be as effective as face-to-face treatment [18,26]. However, guided interventions seem to result in better outcomes [16,27]. Preliminary evidence also suggests that CBIs may be acceptable and effective interventions for adolescents with mental disorders [28,29].

Researchers in the field of eating disorders thus hypothesized that CBIs may also be a suitable approach for eating disorder patients because they mostly come from an age group that uses the computer and Internet frequently. This led them to examine the efficacy of CBIs for eating disorders. There are some reviews of CBIs in the field of eating disorders, but most of them do not meet the requirements of a systematic review [30-33]. To date, Myers et al [34] and Marks et al [35] have presented comprehensive reviews on the use of new technologies in the treatment of a broad range of eating disorders, but only 5 and 8 studies covered CBIs for AN and BN, respectively. The authors concluded that CBIs are a promising approach for the treatment of eating disorders that should be further explored. Dölemeyer et al [36] presented a meta-analysis including 8 controlled studies evaluating CBIs for eating disorders. Guided programs seemed promising as medium to large effect sizes were found. Bauer and Moessner [37] recently provided a review of randomized controlled trials (RCTs) of CBIs for eating disorders and very useful recommendations for future research.

Nevertheless, these reviews do not give a comprehensive overview of the current state of research in the field of TBIs in eating disorders due to their strict inclusion criteria. Our review addresses the fast changes in new technologies and the growing knowledge and increasing research on TBIs for eating disorders. The aims of this review are to provide a comprehensive and up-to-date picture on the developing field of TBIs for eating disorders, specifically for AN and BN, by including a wider range of study designs beyond just RCTs and to discuss the past findings with respect to both acceptance and efficacy of TBIs for AN and BN. We primarily focus on treatment studies, but also consider studies on prevention, motivation, and on programs for carers of eating disorder patients.

## Methods

We searched Medline and PsycINFO for eligible studies published in English, German, Spanish, Italian, French, or Portuguese up to August 2014. The following search terms were used: “(online* OR internet* OR e-mail* OR email* OR web* OR media* OR computer* OR remote* OR tele* OR virtual* OR “interactive voice response*” OR www OR cd* OR dvd* OR flopp* OR audio* OR video* OR palmtop* OR e-health* OR technolog* OR chat* OR software* OR text-messag* OR “text messag*” OR “internet telephony” OR mobile* OR sms*).” Search terms were combined with (anore* OR bulimi* OR “eating disorder*” OR “disordered eating” OR body*image*) in title. The bibliographies of the retrieved articles were also reviewed.

We included studies that met the following inclusion criteria: (1) technology-based psychological interventions, (2) samples including patients with AN and/or BN according to *Diagnostic and Statistical Manual of Mental Disorders* (*DSM*) criteria (*DSM-III* and *DSM-IV*), persons with body image concerns and disordered eating, subthreshold eating disorders, or carers of patients with eating disorders, (3) outcome data at postintervention and/or follow-up, and (4) sample size of at least N=10 per study. There were no age or study type restrictions.

**Figure 1:**
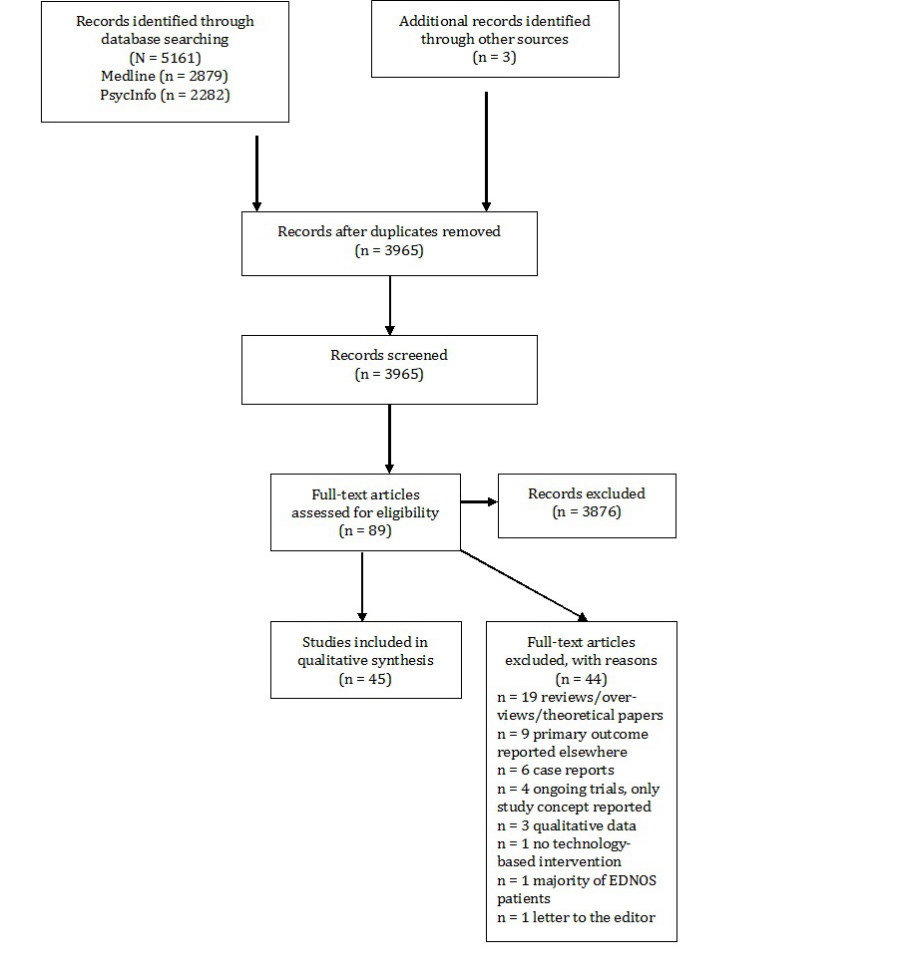
PRISMA flow chart of the literature search.

We excluded psychoeducational or counseling interventions, online support groups, as well as computer-based assessment methods. Studies that evaluated virtual reality in the treatment of body image were excluded because a review covering this issue has already been published [38]. Furthermore, studies evaluating the Internet-based prevention program StudentBodies for eating disorders that generally met our inclusion criteria were excluded because a meta-analysis was recently published on this program [39,40]. Unpublished studies, abstracts of conference proceedings, dissertations, and letters were also not considered. No attempt was made to include unpublished data. Furthermore, inclusion and exclusion criteria were not documented in any official review protocol. Figure 1 shows the flowchart of the literature search.

We separately evaluated CBIs and mobile interventions and divided studies on CBIs into the following main categories: treatment of AN and BN, relapse prevention, prevention and early intervention, and interventions for carers. Treatment studies for AN and BN were further classified into 3 sections depending on the amount of guidance patients received. Studies in which patients were offered no contact with a coach or a clinician were classified as “unguided CBIs.” Studies in which patients worked through programs delivered on a computer or via the Internet and were guided by email, phone, or face-to-face contact with a professional were classified as “guided CBIs.” Finally, interventions that were completely delivered by a therapist (email therapy, videoconferencing) were classified as “therapist-delivered treatments.”

For studies for which relevant data were available, effect sizes (standardized mean differences) were calculated using pooled standard deviations [41]. Within-group effect sizes were calculated between baseline and the following assessment time points (posttreatment, follow-up 1, follow-up 2). Between-group effect sizes at posttreatment and/or follow-up 1 and/or follow-up 2 were calculated. Furthermore, lower and upper confidence intervals for effect sizes were determined [41].

## Results

A total of 40 studies met the inclusion criteria with outcomes reported in 45 publications. There were 41 publications on the results of the efficacy of CBIs (n=21 CBIs for the treatment of eating disorder patients; n=12 programs for prevention and early intervention of eating disorders; n=3 relapse prevention interventions; n=5 interventions for carers of eating disorder patients). A total of 4 publications reported on the efficacy of mobile interventions. Overall, 5 studies focused on AN and 5 studies on adolescents. 22 of the included studies were RCTs, 2 were controlled studies, and the remaining 16 studies were uncontrolled. The studies included a total number of 3646 patients.

Table 1 provides a short overview of included studies. The table in Multimedia Appendix 1 gives detailed information on study characteristics, outcomes, and weaknesses of studies.

In the following, results in effect sizes are given for all categories. Effect sizes and confidence intervals for all individual studies are presented in Multimedia Appendix 2.

**Table 1:** Studies evaluating efficacy of computer- and Internet-based treatment for eating disorders.^a^

Study		Diagnosis (N)	Intervention (study design)	Duration
**Computer- and Internet-based unguided self-help**				
	Bara-Carril et al [42]	BN (N=46); EDNOS (N=11)	“Overcoming Bulimia” (uncontrolled study)	8 modules, 4-8 weeks
	Schmidt et al [43]	BN (N=60); EDNOS (N=37)	“Overcoming Bulimia” (RCT)	8 modules, 8-12 weeks
	Johnston et al [44]	BN (N=94)	Therapeutic writing (RCT)	20 min on 3 consecutive days
**Computer- and Internet-based guided self-help**				
	Huon [45]	BN (N=120)	Internet-guided self-help (RCT)	7 modules, 7 months
	Graham & Walton [46]	BN (N=13); BED (N=27)	“Overcoming Bulimia” (uncontrolled study)	8 modules, 8 weeks
	Murray et al [47]	BN (N=77); EDNOS (N=5)	“Overcoming Bulimia” (controlled study)	8 modules, 8-12 weeks
	Sánchez-Ortiz et al [48]	BN (N=39); EDNOS (N=37)	“Overcoming Bulimia” (RCT)	8 modules, 8-12 weeks; continued access to the online sessions for 24 weeks
	Ljotsson et al [49]	BN (N=33); BED (N=36)	“Overcoming Binge eating” (RCT)	6 modules, 12 weeks
	Carrard et al [50]	BN (N=41); EDNOS (N=4)	“SALUT” (uncontrolled study)	7 modules, 4 months
	Liwowsky et al [51]	BN (N=22)	“SALUT” (uncontrolled study)	7 modules, 4 months
	Nevonen et al [52]	BN (N=27); EDNOS (N=11)	“SALUT” (uncontrolled study)	7 modules, 6 months
	Fernández-Aranda et al [53]	BN (N=62)	“SALUT” (controlled study)	7 modules, 4 months
	Carrard et al [54]	BN (N=100); EDNOS (N=27)	“SALUT” (uncontrolled study)	7 modules, 4 months
	Wagner et al [55]	BN/EDNOS (N=155)	“SALUT” (RCT)	7 modules, 4-7 months
	Leung et al [56]	ED (N=280)	“SMART EATING” (uncontrolled study)	6 components, open-end
	Pretorius et al [57]	BN (N=61); EDNOS (N=40)	“Overcoming Bulimia” (adapted for adolescents) (uncontrolled study)	8 modules, 3 months
	Wagner et al [58]	BN (N=126)	“SALUT” (RCT)	7 modules, 4-7 months
**Internet-based therapist-delivered treatment**				
	Robinson & Serfaty [59]	BN (N=18); BED (N=4); EDNOS (N=1)	Email therapy (uncontrolled study)	3 months
	Robinson & Serfaty [60]	BN (N=51); EDNOS (N=20); BED (N=26)	Email therapy (RCT)	3 months
	Simpson et al [61]	BN (N=5); AN (N=1); EDNOS (N=6)	Videoconferencing (uncontrolled study)	12-20 sessions CBT; 6-8 optional sessions of nutritional education
	Mitchell et al [62]	BN (N=71); EDNOS (N=57)	Videoconferencing (RCT)	20 sessions manual-based CBT, 4 months
**Internet-based relapse prevention**				
	Fichter et al [63,64]	AN (N=258)	“VIA” (RCT)	9 modules, 9 months
	Mezei et al [65]	BN/EDNOS/BED/ AN binge-purging subtype (N=39)	“EDINA” (uncontrolled study)	Online platform for peer support and professional consultation, 4 months
**Body image and eating disorder prevention**				
	Gollings & Paxton [66]	Body dissatisfaction and disordered eating (N=40)	“Set Your Body Free” (pilot RCT)	Weekly 90-min group sessions, 8 weeks
	Paxton et al [67]	Body dissatisfaction (N=116)	“Set Your Body Free” (RCT)	Weekly 90-min group sessions, 8 weeks
	Stice et al [68,69]	Body dissatisfaction (N=107)	“eBody Project” (RCT)	6 modules, 3 weeks
	Serdar et al [70]	Weight and/or shape concerns (N=333)	Dissonance-based eating disorder prevention (RCT)	3 sessions for 60 min
	Zabinski et al [71]	Weight concerns (N=60)	Synchronous Internet relay chat (RCT)	Weekly (60 min) chat discussions, 8 weeks
	Ohlmer et al [72]	Women at risk for AN (N=36)	“Student Bodies” for AN (uncontrolled study)	Weekly sessions for 45-90 min, 10 weeks
	Heinicke et al [73]	Body image or eating problems (N=73)	“My Body, My Life” (RCT)	Weekly 90-min online sessions, 6 weeks
**Eating disorder symptoms/subthreshold eating disorders**				
	Ruwaard et al [74]	Bulimic symptoms (N=105)	Online cognitive behavioral treatment (RCT)	20 weeks
	Jacobi et al [75]	Subthreshold eating disorder (N=29); Eating disorder symptoms at lower level (N=97)	“Student Bodies+” (RCT)	8 sessions, 8 weeks
**Motivation**				
	Hötzel et al [76]	Eating disorder symptoms (N=212)	“ESS-KIMO“ (RCT)	Weekly online sessions for 45 min, 6 weeks
	Leung et al [77]	Eating disorder (N=185)	“SMART EATING” (uncontrolled study)	11 worksheets to enhance individuals’ motivation to change their eating behaviors
**Carers/Parents**				
	Binford Hopf et al [78]	Parents of AN patients (N=13)	Internet-based chat support groups (uncontrolled study)	15 weekly online chat sessions for 90 min
	Grover et al [79]	Carers of AN patients (N=27)	“Overcoming anorexia online” (uncontrolled study)	9 workbooks (encouraged 1 per week, but no formal time limit)
	Grover et al [80]	Carers of AN patients (N=64)	“Overcoming anorexia online” (RCT)	8 modules, 4 months
	Hoyle et al [81]	Carers of AN patients (N=37)	“Overcoming anorexia online” (RCT)	7 modules + 2 additional modules for carers, 7 weeks
	Bruning Brown et al [82]	Parents of sophomore students (N=69)	“Student Bodies” parent intervention (RCT)	Unstructured web-based intervention, 4 weeks
**Mobile/SMS text messaging**				
	Shapiro et al [83]	BN (N=31)	Text messaging (uncontrolled study)	Daily, 24 weeks + 12 CBT face-to-face group sessions for 90 min
	Robinson et al [84]	BN (N=21)	SMS-based intervention (uncontrolled study)	Weekly, 6 months
	Bauer et al [85]	BN (N=97); EDNOS (N=68)	Aftercare SMS-based intervention (RCT)	Weekly symptom report via SMS text message, 16 weeks
	Cardi et al [86]	AN (N=18); BN (N=13)	Mp4 player or iPod with 10 video clips (vodcasts) (uncontrolled study)	Between 3 to 20 min each, workbook and daily monitoring forms, 3 weeks

^a^ AN: anorexia nervosa, BN: bulimia nervosa, BED: binge eating disorder, CBT: cognitive behavioral therapy, ED: eating disorder, EDNOS: eating disorders not otherwise specified, RCT: randomized controlled trial, SMS: short message service.

### Efficacy of Computer- and Internet-Based Interventions for Anorexia Nervosa and Bulimia Nervosa

#### Efficacy of Computer- and Internet-Based Unguided Self-Help

A total of 3 studies with 248 participants including 94 controls examined unguided CBIs for eating disorders. Unguided CBIs showed no effects [44] using the total score of the Bulimic Investigatory Test-Edinburgh (BITE) [87] or small effects [43] using the global score of the Eating Disorder Examination (EDE) [88] from pre- to posttreatment. Furthermore, from preintervention to follow-up, small (EDE global, BITE total) [43,44] to medium effects (binging, vomiting) [42] were found. There was no difference between control groups and unguided CBIs for EDE global or BITE total at posttreatment [43,44], and no (BITE total) [44] to small (EDE global) [43] between-group effects at follow-up.

#### Efficacy of Computer- and Internet-Based Guided Self-Help

##### Adults

Five different treatment approaches of guided CBIs for adults were evaluated in 12 publications. A total of 1051 participants were included. Overall, 135 participants were controls without intervention or on the waiting list; 56 were active controls. From pre- to posttreatment, small to large effects were found for binging and vomiting (in 1 study there was no effect for vomiting) and medium to large effects for eating disorder psychopathology (total score of the Eating Disorder Inventory-2 [EDI-2] [89], EDE global) [48,49,54-56]. From baseline to follow-up treatment, small to large effects were reported for binging, small to medium effects for vomiting, and medium to large effects for eating disorder psychopathology (EDI-2 total, EDE global) [48,52,55]. No differences were observed between guided bibliotherapy and guided CBI both at postintervention and at follow-up (binging, vomiting, EDI-2 total) [55]. Compared to a waiting list control, there were small to medium effects for binging, small to large effects for vomiting, and large effects for EDE global at postintervention [48,49]. At follow-up, there was a medium effect for binging, a small effect for vomiting, and a large effect on EDE global [48].

##### Adolescents

There was 1 study evaluating the potential of CBIs for adolescents and 1 additional study that provided data in the context of a subgroup analysis comparing adult and adolescent patients receiving guided CBI. A total of 130 adolescents were included. Adolescents were compared to 97 adults in 1 study. Small to large effects for binging and medium to large effects for vomiting at both postintervention and follow-up were found [57,58]. Furthermore, medium (EDI-2 total) to large (EDE global) effects were reported for eating disorder psychopathology [57,58] at both time points. In comparison to adults, small effects for binging and vomiting, but no effect for EDI-2 total was shown at postintervention. At follow-up, 1 study showed a medium effect for binging as well as small effects for vomiting and for EDI-2 total [58].

#### Efficacy of Internet-Based Therapist-Delivered Treatments

Four studies examined the efficacy of Internet-based therapist-delivered treatments: 2 investigated email therapy and 2 evaluated videoconferencing. In all, 260 patients participated of whom 27 were waiting list patients and 100 active controls. Internet-based therapist-delivered treatments resulted in medium (BITE severity and symptoms, binging) [59, 62] to large effects (vomiting) [62] from pre- to posttreatment. From preintervention to follow-up, medium (binging) to large (vomiting) effects were found [62]. In comparison to a face-to-face treatment, the effects for binging and vomiting at posttreatment were small and zero at follow-up [62].

#### Efficacy of Internet-Based Relapse Prevention

There were 2 studies investigating the efficacy of 2 different Internet-based relapse prevention programs: 1 uncontrolled study and 1 RCT. A total of 297 patients were included, of whom 130 were treatment as usual (TAU) patients. Overall, small short-term effects were found for the global of the Eating Disorder Examination Questionnaire (EDE-Q) [90] and EDI-2 total [63,65]. No effects were shown for binging, vomiting, and body mass index (BMI) [63,65]. At follow-up, there was a small effect for BMI for completers. Compared to TAU, small effects were found for BMI (completers) and EDI-2 total at postintervention.

### Efficacy of Internet-Based Treatments for Prevention and Early Intervention

#### Efficacy of Internet-Based Treatments for Body Image Problems and Prevention of Eating Disorders

##### Adults

Seven studies investigated the efficacy of CBIs in the prevention of eating disorders. A total of 775 participants were included: 224 were controls without intervention, 207 active controls (face-to-face), and 49 video or brochure controls. Primary outcomes studied were mainly body dissatisfaction and thin-ideal internalization. From pre- to postintervention, there were small to medium effects for the Ideal-Body Stereotype Scale-Revised (IBSS-R [91]) [70], as well as medium to large effects for the Body Shape Questionnaire (BSQ [92]) [66,67] and for EDE-Q global [71,72]. From baseline to follow-up, there was a small effect for IBSS-R [69], medium effects for EDE-Q global [71,72], and a large effect for BSQ [66]. At posttreatment, no (IBSS-R) [68,70] to small effects (BSQ) [66,67] of prevention programs were seen in comparison to face-to-face conditions, small (EDE-Q global, IBSS-R) [70,71] to medium effects (BSQ) [67] in comparison to controls without intervention, and medium to large effects (IBSS-R) [68] in comparison to a video and a brochure control condition, respectively [68]. At follow-up, no (BSQ) [66] to small effects (IBSS-R) [69] were observed in comparison to face-to-face conditions, a small effect in comparison to a control condition (EDE-Q global) [71], and no effects in comparison to a video as well as a brochure control condition (IBSS-R) [69].

##### Adolescents

Heinicke et al [73] reported a medium effect for an adolescent sample from pre- to posttreatment in BSQ that was maintained at follow-up. They also found a medium between-group effect in comparison to a delayed treatment group.

#### Efficacy of Internet-Based Treatments for Subthreshold Eating Disorders

We identified 2 studies investigating Internet-based treatments for subthreshold eating disorders. A total of 231 participants were randomized, of whom 35 were active controls and 97 were on a waiting list. From pre- to postintervention, a medium effect for binging [75], a small effect for vomiting [75], and medium to large effects for EDE-Q global [74,75] were observed. From baseline to follow-up, large effects for binging [75] and EDE-Q global [74,75], but no effect for vomiting [75] was identified. At postintervention, the Internet intervention differed only slightly from bibliotherapy (EDE-Q global) [74]. In comparison to a waiting list, there were small effects for binging and vomiting [75], and no effect in 1 study, but a medium effect for EDE-Q global in another study [74,75]. At follow-up, no differences were found in comparison to bibliotherapy (EDE-Q global) [74]. Compared to the waiting list, there were small effects for binging and vomiting and a medium effect for EDE-Q global [75].

#### Efficacy of Internet-Based Treatments to Enhance Motivation to Change in Eating Disorders

Internet-based treatments that aimed at enhancing motivation to change in eating disorders were the subject of 2 studies. A total of 397 participants were included, of whom 109 were waiting list controls. There were small time effects for binging [77] and motivation to gain weight in problematic areas [76], but no time effects for vomiting [76,77] or for motivation to give up binging and vomiting [77] from baseline to postintervention. At follow-up, there was a small effect for the motivation to give up binging, but no effects for binging, vomiting, or motivation for giving up vomiting [77]. A small between-group effect in vomiting was reported, but no between-group effect for motivation to gain weight in problematic areas in comparison to a control group at posttreatment.

#### Efficacy of Internet-Based Treatments for Carers of People with Eating Disorders

There were 5 intervention studies including 210 carers of people with eating disorders. A control intervention called Beating Eating Disorders (BEAT; patient and carer organization) was offered to 30 carers, whereas 47 received no intervention. From baseline to postintervention and to follow-up, no effect was found for the Level of Expressed Emotion Scale (LEE [93]) (unguided overcoming anorexia online [OAO]), a small effect was found for LEE (guided OAO) [81], and a medium effect was found for the total score of the Hospital Anxiety and Depression Scale (HADS [94]) (guided OAO) [79]. Furthermore, medium effects for the scales “critical to others” and “healthy outlook” of the Parental Attitudes and Criticism Scale (PACS [82]) were seen at postintervention, but only small effects at follow-up [82]. Finally, a large effect for the total score of the Eating Disorder Symptom Impact Scale (EDSIS [95]) was evident at postintervention [78]. In comparison to a control group, there were small effects for “critical to others” and “healthy outlook” at postintervention [82]. Between unguided and guided OAO, a small effect was shown at postintervention and a medium effect at follow-up [81].

#### Efficacy of Mobile Therapy in the Treatment of Eating Disorders

Four studies evaluated mobile interventions for eating disorders. Three studies (total N=230; n=83 control patients) employed short message service (SMS) text messaging and 1 study used vodcasts (N=31). For SMS text messaging interventions, the baseline versus follow-up effects were small [84] to large [83] for binging, zero [84] to medium [83] for vomiting, and large for EDI-2 total [83]. Until follow-up, a large effect for binging, a medium effect for vomiting, and a large effect for EDI-2 total [83] were reported. For the vodcast intervention, no effect was found for binging and small effects for vomiting and EDE-Q total [86].

### Abstinence Rates for Technology-Based Interventions

Abstinence rates ranged between 12% [43] and 46.2% [42] for unguided CBIs (waiting list: 10% to 20% [43]), between 16.6% [45] and 46.6% [45] for guided CBIs (waiting list: 0% [45] to 20.7% [48]), and between 21% [62] and 66.7% [61] for therapist-delivered treatments (waiting list: 0% [60]; face-to-face: 25.8% to 53.3% [62]). In studies evaluating CBIs for subthreshold eating disorders, abstinence rates were between 37% [74] and 45.1% [75] (waiting list: 8% [74] to 26.9% [75]; bibliotherapy: 8% to 34% [74]). Mobile interventions showed abstinence rates between 29.4% [84] and 37.8% [85] (control: 18.1% [85]). Abstinence rates for individual studies are shown in Multimedia Appendix 3.

In the following, results of further relevant aspects of TBIs are presented. Note that not all studies mentioned so far provided data regarding these points.

### Intervention Uptake

Twenty studies provided information about non-take-up rates. These ranged from 2.9% [80] (carers) to 50% [82] (carers) with an average of 20.1%. However, reasons for non-take-up were rarely reported. Paxton et al [67] and Hoyle et al [81] indicated that persons did not initiate treatment due to technical difficulties or difficulties accessing a computer or the website, due to seeking alternative treatment, or due to not having enough time to take part in the intervention.

Murray et al [47] found no differences between those who started the program and those who did not, except for the expected personal usefulness of this kind of intervention. In the study by Leung et al [56], patients who took up the intervention had a lower BMI and were generally more educated. There were no differences in age or severity of eating disorder. Leung et al [77] reported that participants working through the motivational enhancement exercises had a slightly higher stage of change than those who did not take part in these.

### Acceptance

Twenty-six studies provided data about compliance rates (we defined compliance as the full completion of the intervention regardless of whether participants completed posttreatment and/or follow-up assessment, and we only extracted data from studies that explicitly reported information on this). Between 18.4% [52] and 95.5% [51] completed the program (mean compliance rate in the TBI conditions: 57.6% of patients that were included for uncontrolled studies or RCTs).

Pretorius et al [57] found that higher baseline scores on eating concerns were associated with higher compliance in terms of number of completed sessions. Fichter et al [63] reported that patients with less pronounced compensatory behaviors and a lifetime mood disorder showed better adherence. The results by Leung et al [56] suggested that the motivational stage of change in patients and their treatment expectations predict compliance particularly well. These authors also named reasons for discontinuing: motivational difficulties, not enough energy and time for the program, loss of interest, or nonresponse.

Most of the studies found no differences between completers and noncompleters in sociodemographic or clinical variables or in baseline scores [55,66,67,73,75,77,84]. Serdar et al [70] reported that noncompleters had higher baseline scores in thin-ideal internalization. Ljotsson et al [49] found that completers showed less bulimic episodes at baseline. Likewise, Carrard et al [50] showed that completers and noncompleters differed in frequency of binges and vomiting.

Satisfaction was either measured by a satisfaction scale or assessed by qualitative comments. The majority of participants were satisfied with the programs and rated modules as pleasant, easy, and useful [46,71,72,75,78,96]. Patients also said that they would recommend the programs to others [54,72,74,78,97]. Some patients stated that they preferred participating in a program offered via the Internet over face-to-face treatment [71,73]. However, there were also patients who would recommend it only as an adjunct to face-to-face psychotherapy [52].

### Follow-Up Dropouts

Follow-up dropout rates in the studies included in this review ranged from 4.7% [69] to 84.8% [46]. In terms of predictors of dropout, results were inconsistent. There were studies where no predictors could be identified [43], whereas others found that dropout could be predicted by higher anxiety scores, a lower hyperactivity, a lower minimum BMI, and lower reward dependence scores on the Temperament and Character Inventory-Revised (TCI-R [98]) [53]. Graham and Walton [46] reported that dropouts were characterized by higher scores on the “drive for thinness” EDI-3 scale. Fichter et al [63] observed that lack of time or not wanting to deal with the eating disorder prevented patients from taking part in the follow-up measurement. Bauer et al [85] found that follow-up dropouts did not differ from those who completed follow-up assessment in any of the baseline characteristics.

### Predictors of Outcome

Several studies reported predictors of outcome of TBIs in eating disorders. A better state of general psychological health was found to predict a better outcome [54]. In relation to primary symptomatology, Marrone et al [99] identified reduction of binge eating as the best indicator of a positive treatment outcome (abstinence) at the 1-year follow-up, whereas for short-term follow-up (3 months), it was the reduction of purging behavior. Moreover, higher scores on the EDI “perfectionism” scale and on the Eating Attitudes Test (EAT) [100], as well as a higher minimum BMI significantly correlated with a better outcome [53]. Johnston et al [44] reported that patients who benefited the most from their intervention were those with high body shame at baseline. Fichter et al [63] found lower scores on the Structured Interview for Anorexic and Bulimic Syndromes (SIAB-EX [101]) “compensatory behavior” subscale and higher spontaneity to be predictive of successful relapse prevention. Mezei et al [65] identified a higher ratio of words related to family of origin, a higher BMI, and lower binging and emotional distress at baseline to be significantly associated with change in EDE-Q total. Furthermore, better compliance was associated with a better outcome also. Adherence and more spontaneity were also identified as long-term predictors for a favorable course, as were higher scores on the “ineffectiveness” scale of the EDI-2. In the “SALUT” program, improvement increased with the number of diary entries and the number of steps patients completed [50,54]. Even in email therapy, patients who displayed more commitment in terms of number of words written had better outcomes [59,60]. Paxton et al [67] and Serdar et al [70] also found a correlation between the number of sessions and outcome, whereas Zabinski et al [71] found no such correlation. Finally, Cardi et al [86] reported that the mean use of the vodcasts was correlated with the BMI change.

### Details About Guidance

Six eating disorder studies presented information about therapists’ time and efforts in guided CBIs. It ranged between 45 minutes and 135 minutes per patient [47,79,80,86,102]. Therapists in the study by Leung et al [56] invested approximately 5-10 minutes per email. The intervention study of Ruwaard et al [74] included 25 scheduled therapist feedback moments, which took approximately 13 hours to complete. Regarding content, emails from therapists were predominantly supportive (95.4%) with only 14.7% of the emails containing at least 1 CBT comment and 13.6% at least 1 technical comment [102].

### Therapeutic Alliance

Only 1 study on videoconferencing for eating disorder patients investigated therapeutic alliance. Therapists experienced differences between the delivery methods in terms of adherence to therapeutic tasks, adherence to therapeutic goals, and therapeutic bond, whereas patients did not [103].

### Cost-Effectiveness

Only 1 study compared cost-effectiveness of face-to-face CBT and CBT delivered via telemedicine [104]. Efficacy was found to be comparable, whereas costs of telemedicine were lower, although still substantial.

### Risks and Side Effects of TBIs

Three studies provided information about adverse events occurring in the reviewed TBI studies. Sánchez-Ortiz et al [48] as well as Robinson and Serfaty [60] reported that no major adverse events occurred in their studies. Fichter et al [63] reported that 11 of 258 (4.3%) AN patients showed adverse events (BMI<13.5 during the course of the trial). Fortunately, no serious adverse event (acute suicidality, suicide attempts, or death) was identified.

## Discussion

### Principal Findings

Interest in research on TBIs for AN and BN has increased during the last decade. Forty studies whose outcome results were published in 45 papers fulfilled the inclusion criteria of this systematic review. Most studies dealt with guided CBIs (“Overcoming Bulimia,” “SALUT”) or programs for the prevention and early intervention of eating disorders. One has to consider that many of these treatments are derived from (guided) self-help interventions based on other media (eg, self-help manuals such as “Overcoming Bulimia”) that have been evaluated by a considerable number of researchers in the past and whose results have been summarized in a current meta-analysis [105]. Other approaches were rarely investigated: only 2 studies looked into videoconferencing and email therapy. Merely 3 studies evaluated unguided CBIs and 2 researched the potential of CBIs in relapse prevention.

### Summary of Main Results

With regard to BN, guided CBIs led to improvements in the core symptoms of binging and purging and global eating disorder psychopathology. Patients receiving guided CBIs improved more than controls. Guided CBI was shown to be as effective as guided bibliotherapy. Initial findings suggest that treatment results can also be maintained at follow-up. Furthermore, videoconferencing showed promise in treating patients with BN. Unfortunately, this approach has only been evaluated in 1 RCT so far.

With regard to AN, CBIs might be used for relapse prevention. However, only 1 study has empirically evaluated this kind of intervention in AN patients so far. Several case reports by Yager [106-108] suggest that CBIs (eg, email) may be effective when used as a therapeutic adjunct in the outpatient treatment of AN. In his opinion, CBIs increase adherence, patient satisfaction, and therapeutic alliance, and he did not observe any negative effects. Therefore, he suggests including email contact in the regular therapy process to enhance weekly sessions, to monitor daily food intake, or for crisis intervention.

With regard to adolescents with eating disorders, research findings suggest that CBIs may be a treatment option for bulimic patients. However, results should be confirmed in RCTs and replicated by other research groups before widely recommending it.

Furthermore, CBIs may also be considered in the prevention and early intervention of eating disorders as well as for supporting carers of eating disorder patients. Finally, preliminary evidence suggests that mobile interventions are useful for patients with eating disorders in relapse prevention or as an adjunct to therapy (eg, symptom monitoring).

Efficacy results of TBIs are in-line with studies evaluating CBIs in other mental health disorders. For example, medium effects at posttreatment were also shown in a meta-analysis of CBIs for depressive disorders [16]. Videoconferencing—the intervention that is closest to traditional face-to-face therapy—showed very promising results. Videoconferencing was also shown to yield similar clinical outcomes as face-to-face therapy with regard to a variety of other disorders [109]. Although this intervention provides no benefit for the therapist in terms of time or cost-effectiveness, it might be an attractive alternative treatment for patients living far away from a specialist eating disorder therapist. CBIs for carers of eating disorder patients are a relatively new field of research. In-line with Hu et al [110] who showed that Internet-based interventions were able to reduce stress and to improve well-being of caregivers for others with medical conditions, we found that CBIs may also be helpful for carers of patients with eating disorders.

Although efficacy results in the reviewed studies are promising, high rates of non-take-up, noncompliance, and dropout in the reviewed studies severely hamper the validity of study results. Therefore, findings must be interpreted with some caution. The result that only slightly more than half of the participants (57%) completed the offered TBI parallels the report by Waller and Gilbody [111] that only half of patients completed a full course of CBI for anxiety and depression. However, as adherence and satisfaction are important determinants of therapy outcome, it will take additional efforts to improve acceptance of TBIs for participants. This may be accomplished by educational advertising and by developing specific, individually tailored programs that appeal to participants searching for help. To increase adherence, future interventions should feature the following characteristics: a strong theoretical foundation, perceived personal relevance, tailoring, persuasive technologies, credibility, social networking, and common “push factors,” including human support and/or periodic prompts (by email or telephone), as suggested by Murray [112].

### Is Human Contact and Guidance Necessary?

Guidance may be essential for both compliance and outcome of CBIs [113,114]. In the field of research on CBIs for eating disorders, only 3 studies directly compare unguided and guided CBI. Huon [45] found more improvement in the 2 guided groups. In contrast, Murray et al [115] did not find any significant differences between brief therapist guidance and minimal researcher guidance. However, this study was not an RCT, but rather compared the results of 2 consecutive cohorts. Hoyle et al [81] got mixed results on whether further guidance is useful. Eating disorder patients see guidance as a helpful and important element of the intervention [54,97].

Studies of CBIs for various mental health disorders showed that guidance augments efficacy [27]. Palmqvist et al [114] calculated a meta-analytic correlation of ρ=.75 between time invested by therapists and outcome. Outcomes equivalent to face-to-face therapy were also reported [116,117].

Overall, details about guidance are unsatisfactorily reported in most studies on CBIs for eating disorders. Information about the type and qualifications of coaches, about the type of support, as well as the timing, frequency, and overall amount of contact is often missing. However, all this important information is required to be able to compare the different programs and their efficacy and to get a clearer picture of how much and what kind of contact is needed to optimize the interventions.

### Therapeutic Alliance

The issue of human contact in guided CBIs also raises concerns about whether online therapy can establish any meaningful therapeutic alliance [118]. The only study in the field of CBIs for eating disorders suggests that videoconferencing establishes an adequate therapeutic alliance [113]. However, there are no findings regarding its quality when nonverbal cues are completely lost, such as in email guidance.

A systematic review investigating therapeutic relationships in e-therapy for mental health suggests that a therapeutic alliance equivalent to that in face-to-face therapy can be established in Internet-based therapy [119]. However, this topic is underresearched because just 11 (1.3%) of 840 reviewed studies reported data on this matter. Furthermore, Andersson et al [120] speculated that despite high alliance ratings, these relationships may not be as important as in face-to-face therapy. In conclusion, much more research is necessary to really understand similarities and differences as well as special features of therapeutic relationships in both delivery methods.

### Strengths and Limitations of This Review

One strength of the present review is that we considered the whole spectrum of care from prevention, early intervention, treatment, relapse prevention of eating disorders, to interventions for carers of eating disorders. Furthermore, by including a wide range of study designs beyond just RCTs, a broad range of new types of TBIs were included. This made it possible to give a comprehensive up-to-date picture of the dynamic field of TBIs in eating disorders. Moreover, a wide range of aspects relevant to TBIs, such as acceptance, efficacy, predictors of outcome, need of guidance, and therapeutic alliance, were reviewed. This review is limited by the fact that a meta-analysis could not be performed due to the enormous heterogeneity of studies. Furthermore, diagnoses in studies were made in sometimes more but also less rigorous ways (informal clinical interview, semistructured clinical interview, questionnaire using *DSM* criteria), thereby reducing the chances that all studies had the same diagnostic threshold. Unfortunately, no pure samples of BN patients were available in most studies. Instead, patients with EDNOS or binge eating disorder were also included, so that results must be interpreted with some caution.

There are also several methodological limitations in the studies discussed in this review. We mainly followed the coding for weaknesses as suggested by Newman et al [121] (see table in Multimedia Appendix 1). Sixteen studies were uncontrolled and overall, 55% RCTs with either a control group or 13 with an active treatment as a comparator were still too few. For 11 studies, no follow-up data were available and in 28 studies outcomes were determined only through self-report and not by blinded interviewers. Sample sizes were predominantly moderate and rarely included more than 100 participants; 14 studies had sample sizes of less than 50. In most cases, no power calculation was reported. Fifteen studies did not report on additional psychological treatment, as did 22 studies on pharmacological treatment. Twenty-two studies failed to present information on adherence or quality checks in the treatment as did 18 studies on therapist training regarding TBIs. Most studies did not explicitly investigate outcome predictors or the active ingredients of mostly complex interventions. Outcome measures varied considerably making it hard to compare different study results.

Consequentially, a number of challenges for future research arise that are detailed subsequently.

### Recommendations for Future Research

There is need for more high-quality RCTs that adhere to the Consolidated Standards of Reporting Trials (CONSORT) statement [122], with sufficient sample sizes and long-term follow-ups to investigate stability of effects. Future studies should compare TBIs for AN and BN patients not only concerning waiting list or no treatment comparisons, but also with respect to active conditions such as face-to-face psychotherapy and traditional (manual-based) self-help interventions. Furthermore, different modes of TBIs as well as TBIs with different amounts of guidance should be compared with each other to derive information about comparative effectiveness. Future research should also evaluate the helpfulness of TBIs in preparing patients for face-to-face therapy, and explore the potential of combining face-to-face therapy and TBIs. Outcomes of evaluations should be preferably assessed by independent, blinded assessors and not by self-reports alone. To facilitate comparability of treatments, standardized outcome measures should be used. In addition, abstinence and compliance should be defined consistently across studies. Furthermore, reporting of studies should be improved. Studies should include information about additional psychological and/or pharmacological treatments to control for outside effects. Additionally, information about adherence or quality checks on treatment needs to be consistently reported. In addition, detailed descriptions of guidance should be given including information on qualification and on training of the coach/therapist. Modality, frequency, and intensity of guidance, as well as information about therapist’s time and efforts should also be specified. To improve the methodological quality and the reporting of studies, researchers should follow the guidelines for executing and reporting Internet intervention research [123].

Many questions regarding the optimal delivery method of TBIs, as well as the optimal dose in terms of frequency, intensity, and duration of interventions, still await qualified answers. Research shows that adding more therapy components does not lead to better results [124]. In this context, Andersson and Titov [125] pointed out that it is important to consider patients’ capacity and to avoid overloading. Studies should also try to find out how much time patients have to spend on a treatment program to achieve maximal benefits [24]. Similarly, questions regarding optimal guidance remain unresolved: research needs to identify the usefulness of clinical contact [24] and the ideal frequency and kind of provided support [125]. The issue of what background the therapist should ideally have and how much face-to-face contact a therapist needs to have in order to be empathetic, interested, present, and thus therapeutically effective, also needs clarification [126]. In case of engaging TBIs of adequate quality, therapist expertise may not be as important as in face-to-face therapy [125]. Furthermore, future studies should also include checking adherence to the intervention protocol of therapists who provide guidance and the quality of their guidance.

Usually TBIs are complex interventions (ie, they consist of a number of interacting components) [127]. Therefore, it is essential to explore mechanisms of action of TBIs and how therapy can be optimized by identifying the active ingredients. In addition, mediators and moderators of therapeutic change in TBIs should be explored. Future research will also have to clarify who benefits, who drops out, and what factors increase compliance. Moreover, the impact of sociodemographic factors, such as sex, age, and/or education, needs to be elucidated.

Integrating more motivational components into programs and tailoring the treatment to the AN and BN patients’ needs in terms of severity of illness and comorbidity will be imperative to further improve interventions. Research on depression has shown that more severely affected patients respond better to tailored, rather than to nontailored treatment [128]. Patients’ preferences should also be considered when designing future interventions (eg, by providing a choice of different treatment modules). Therefore, it is necessary to conduct qualitative studies to explore patients’ opinions and wishes concerning TBIs and to explore acceptance.

More research on how to enhance the uptake and utilization of TBIs and how to promote its broader dissemination and implementation in routine care is necessary. TBIs should be integrated into existing health care systems and efforts should be made to enhance their acceptability and adoption by patients as well as by health care delivery teams [129]. Many patients and clinicians still adopt a skeptical attitude toward TBIs, a problem that can be addressed through education [125]. More pragmatic RCTs in naturalistic settings should be performed to evaluate effectiveness and cost-effectiveness of TBIs within a stepped-care program [130]. This kind of research is able to identify where TBIs fit best into existing treatment options and to economically integrate them into our health care system [129]. TBIs may be presented as a first step in a stepped-care process probably followed by or combined with other treatment options such as face-to-face psychotherapy [125]. Cavanagh et al [131] reported a naturalistic nonrandomized trial of CBI for anxiety and/or depression that found CBI to be effective even under routine conditions, suggesting good generalizability.

TBIs also have some disadvantages. Some patients may not be able to access technological interventions [132] or may feel uncomfortable using them [129] due to security issues [125] and privacy concerns [133]. Moreover, the lack of nonverbal cues (eg, in email therapy) can lead to a loss of important therapeutic information and to misinterpretations. Additionally, the lack of immediate exchange may cause a patient who expects immediate response to feel insecure and helpless [133]. Furthermore, patients’ safety cannot be guaranteed and, in case of a crisis, there are few options for action. Kiluk et al [134] advocate that future studies should focus more on potential adverse events in TBIs before a final recommendation for TBIs is made. So far, adverse events have rarely been considered in reports of TBIs for eating disorder patients. Therefore, this issue should be more systematically addressed. In addition, future studies should focus on the identification of nonresponders or patients that even deteriorate (eg, by symptom monitoring on a regular basis) and on the development of treatment strategies for these patients.

Last but not least, research needs to determine the limits of TBIs [126]. In-line with Proudfoot et al [135], TBIs for eating disorders may not be indicated in case of a BMI<17.5, severe vomiting, comorbid posttraumatic stress disorder, severe depression, or a psychotic illness.

### Conclusions

Unfortunately, the evidence for TBIs in the treatment of AN and BN remains insufficient because many approaches were investigated only once, by 1 research group, or only in uncontrolled studies. Nevertheless, the initial results are encouraging. At this stage, unguided CBIs cannot be recommended for the treatment of AN and BN, whereas guided CBIs may be a promising treatment approach, especially for BN. Videoconferencing may also be an approach worth pursuing further in research as well as in practice. Furthermore, Internet-based relapse prevention for AN inpatients may be an effective way to stabilize treatment success and to bridge the gap between inpatient and outpatient therapy. Guided CBIs seem to be a promising approach even in the treatment of adolescents with BN. Efficacy of email therapy still remains to be seen. CBIs might also be considered for the prevention of eating disorders as well as to support carers of eating disorder patients. Furthermore, evaluation of mobile interventions should be further pursued.

Until now, many TBIs have only been used within the context of research studies and have not become part of routine health care. Before a widespread implementation of TBIs, it is imperative to ascertain that they are also feasible under naturalistic conditions and across settings.

In conclusion, one should stay open-minded about the integration of novel technologies that may enhance psychological prevention and treatment of AN and BN and their carers. TBIs can especially serve as a first step in a stepped-care model. However, patient compliance, which is essential for TBIs to work, is still a major challenge. Future research is needed before widely recommending TBIs for AN and BN.

## Multimedia Appendix 1

Studies evaluating efficacy of technology-based psychological treatments for anorexia and bulimia nervosa.

## Multimedia Appendix 2

Within- and between-group effect sizes and confidence intervals for included studies (posttreatment and follow-up).

## Multimedia Appendix 3

Definition of abstinence in studies included in the review as well as abstinence rates and results of significance test wherever data was available.

## Abbreviations

AN: anorexia nervosa
BEAT: Beating Eating Disorders
BED: binge eating disorder
BITE: Bulimic Investigatory Test-Edinburgh
BMI: body mass index
BN: bulimia nervosa
BSQ: Body Shape Questionnaire
CBI: computer- and Internet-based intervention
CBT: cognitive behavioral therapy
CONSORT: Consolidated Standards of Reporting Trials
DSM: Diagnostic and Statistical Manual of Mental Disorders
EAT: Eating Attitudes Test
ED: eating disorder
EDE: Eating Disorder Examination
EDE-Q: Eating Disorder Examination Questionnaire
EDI-2/EDI-3: Eating Disorder Inventory-2/Eating Disorder Inventory-3
EDNOS: eating disorder not otherwise specified
EDSIS: Eating Disorder Symptom Impact Scale
HADS: Hospital Anxiety and Depression Scale
IBSS-R: Ideal-Body Stereotype Scale-Revised
IPT: interpersonal psychotherapy
LEE: Level of Expressed Emotion Scale
OAO: Overcoming Anorexia Online
PACS: Parental Attitudes and Criticism Scale
PRISMA: Preferred Reporting Items for Systematic Reviews and Meta-Analyses
RCT: randomized controlled trial
SIAB-EX: Structured Interview for Anorexic and Bulimic Syndromes
SMS: short message service
TAU: treatment as usual
TBI: technology-based interventions
TCI-R: Temperament and Character Inventory-Revised
