# Supplementary material for: The Potential of Technology-Based Psychological Interventions for Anorexia and Bulimia Nervosa: A Systematic Review and Recommendations for Future Research
Source: J Med Internet Res. 2015 Mar 31;17(3):e85. doi: 10.2196/jmir.3554 (PMC4397416; doi:10.2196/jmir.3554)
Supplement: Supplementary file 1 [file jmir_v17i3e85_app1.pdf]

Studies evaluating efficacy of technology-based psychological treatments for anorexia and bulimia nervosa [a]

**Study**	**Participants**		**Recruitment**	**Design/ Assessment timepoints**	**Intervention and conditions**		**Contact**	**Outcome**	**Comp-liance (comple-tion of treat-ment)**	**Weak-nesses of studies**
	**Diagnosis (N, assess-ment tool)**	**Number of partici-pants/ starters (uptake rate)**			**Intervention Duration**	**Conditions N (in case of RCT)**				
**Computer- and Internet-based Unguided Self-help**										
Bara-Carril et al [42]<br><br>United Kingdom	BN (N=46) EDNOS (N=11) (DSM-IV)	57/47 (82.5%)	Consecutive referrals to the ED unit	**Uncontrolled study**<br>Baseline, session 3, session 8 (post), 2-month follow-up	**"Overcoming Bulimia"**<br>8 modules<br>4-8 weeks		No contact with a clinician	Improved	19/57 (33.3%)	1,3
Schmidt et al [43]<br><br>United Kingdom	BN (N=60) EDNOS (N=37) (DSM-IV)	97/64 (66.0%)<br>CD-ROM 49/32 (65.3%)<br>WL 48/32 (66.7%)	Consecutive referrals to the ED out-patients service	**RCT**<br>Baseline, 3 months (post), 4-month follow-up	**"Overcoming Bulimia"**<br>8 modules<br>8-12 weeks	CD-ROM + 5 or 15 F2F-sessions (N=49)<br>Waiting list (WL) + 15 F2F-sessions (N=48)	CD-ROM<br>No practitioner guidance	CD-ROM = WL	CD-ROM 16/49 (32.7%)	4,8
Johnston et al [44]<br><br>United Kingdom	BN (N=94) (BITE >10)	94	Universities, colleges, Internet	**RCT**<br>Baseline, 4-week follow-up, 8-week follow-up	**Therapeutic writing**<br>20 min on 3 consecutive days	Therapeutic writing (N=48)<br>Control writing (N=46)	No guidance	Therapeutic writing = Control writing	Therapeu-tic writing 40/48 (83.3%)<br>Control writing 42/46 (91.3%)	3,5
**Computer- and Internet-based Guided Self-help**										

Huon [45]<br><br>Australia	BN<br>(N=120)<br>(DSM-III)	120	Community	**RCT**<br>Baseline,<br>7 months (post),<br>3-month follow-up,<br>6-month follow-up	**Internet-guided self-help**<br>7 modules<br>7 months	Group 1-3:<br>Mailed program<br>(in each case N=30)<br>Control group<br>(N=30)	Group 1<br>Contact to cured person<br>Group 2<br>Contact to improved person<br>Group 3 & Control group<br>No contact	Group 1/Group2 > Group3 > Control group		3,5,7
Graham & Walton [46]<br><br>United Kingdom	BN<br>(N=13)<br>BED<br>(N=27)<br>(DSM-IV)	77/66<br>(85.7%)	Consecutive referrals to the ED service	**Uncontrolled study**<br>Baseline,<br>session 8 (post),<br>1-year follow-up<br>(not published)	**“Overcoming Bulimia”**<br>8 modules<br>8 weeks		Three scheduled contacts with a clinician	Improved	40/77<br>(51.9%)	1,3,4,6
Murray et al [47]<br><br>United Kingdom	BN<br>(N=77)<br>EDNOS<br>(N=5)<br>(DSM-IV)	103/82<br>(79.6%)<br>MG<br>53/43<br>(81.1%)<br>TG<br>50/39<br>(78.0%)	Consecutive referrals to the ED unit	**Controlled study**<br>Baseline,<br>session 3 (post),<br>6-8-week follow-up	**“Overcoming Bulimia”**<br>8 modules<br>8-12 weeks	Minimal guidance group (MG)<br>(N=53)<br>Therapist guidance group (TG)<br>(N=50)	MG<br>Low-key interaction with a researcher<br>TG<br>Three 20-min sessions of therapist guidance	MG = TG	MG<br>20/53<br>(37.7%)<br>TG<br>17/50<br>(34.0%)	3,4,6,7
Sánchez-Ortiz et al [48]<br><br>United Kingdom	BN<br>(N=39)<br>EDNOS<br>(N=37)<br>(DSM-IV)	76/55<br>(72.4%)<br>iCBT<br>38/30<br>(78.9%)<br>WL<br>38/25<br>(65.8%)	University, email	**RCT**<br>Baseline,<br>3 months (post),<br>3-month follow-up	**“Overcoming Bulimia”**<br>8 modules<br>8-12 weeks<br>continued access to the online-sessions for 24 weeks	Internet-based cognitive behavioral therapy (iCBT)<br>(N=38)<br>Waiting list/Delayed treatment control group (WL)	iCBT<br>Email support once every 1-2 weeks	iCBT > WL	Mean number of sessions<br>iCBT<br>5.5<br>(S.D.=2.5)<br>WL<br>5.3<br>(S.D.=2.5)	6,7,8

						(N=38)				
Ljotsson et al [49]<br><br>Sweden	BN (N=33)<br>BED (N=36)<br>(DSM-IV)	73/69 (94.5%)<br>Inter-vention group<br>37/35 (94.6%)	Community, homepage of anorexia/bu-limia patients association	**RCT**<br>Baseline, weekly reports, 6 weeks (mid-treatment), 3 months (post), 6-month follow-up	**"Overcoming Binge eating"**<br>6 modules<br>12 weeks	Intervention group<br>(N=37)<br>Waiting list (WL)<br>(N=36)	Email-contact with a therapist at least once a week	Intervention group > WL	Interven-tion group<br>24/37 (64.9%)	3,7,8
Carrard et al [50]<br><br>Switzerland	BN (N=41)<br>EDNOS (N=4)<br>(DSM-IV)	45	Community, referrals from local health care professionals	**Uncontrolled study**<br>Baseline, 4 months (post), 2-month follow-up	**"SALUT"**<br>7 modules<br>4 months		Weekly email contact with a therapist	Improved	29/45 (64.4%)	1,3,5,6,7,9
Liwowsky et al [51]<br><br>Germany	BN (N=22)<br>(DSM-IV)	22	Internet, newspaper, public bulletins	**Uncontrolled study**<br>Baseline, 4 months (post), 2-month follow-up	**"SALUT"**<br>7 modules<br>4 months		Weekly email contact with a psycholo-gist	Improved	21/22 (95.5%)	1,4,5,6,7,9
Nevonen et al [52]<br><br>Sweden	BN (N=27)<br>EDNOS (N=11)<br>(DSM-IV)	38	Patients from waiting list at the AN and BN unit	**Uncontrolled study**<br>Baseline, 6 months (post), 2-month follow-up	**"SALUT"**<br>7 modules<br>6 months		Weekly email contact with a coach	Improved	7/38 (18.4%)	1,9
Fernández-Aranda et al [53]<br><br>Spain	BN (N=62)<br>(DSM-IV)	62	Referrals at the Department of Psychiatry	**Controlled study**<br>Baseline, 4 months (post)	**"SALUT"**<br>7 modules<br>4 months	Internet-based therapy (IBT)<br>(N=31)<br>Waiting list (WL)<br>(N=31)	Weekly email contact with a coach	IBT > WL		2,3,4,5,6,78
Carrard et al [54]<br><br>Switzerland<br>Spain	BN (N=100)<br>EDNOS (N=27)<br>(DSM-	127	Internet, newspaper articles, ad-vertisements, study center	**Uncontrolled study**<br>Baseline, 4 months (post), 2-month follow-	**"SALUT"**<br>7 modules<br>4 months		Weekly email contact with a coach	Improved	40/127 (31.5%)	1,3,5,6

Sweden<br>Germany	IV)		waiting list, referrals to the study center	up						
Wagner et al [55]<br><br>Austria<br>Germany	BN or EDNOS (N=155) (DSM-IV)	155/126 (81.3%)<br>INT-GSH 83/70 (84.3%)<br>BIB-GSH 72/56 (77.8%)	Advertise-ments	**RCT**<br>Baseline,<br>4 months,<br>7 months,<br>18 months	**“SALUT”**<br>7 modules<br>4-7 months	Internet-based guided self-help (INT-GSH) (N=70)<br>Conventional guided self-help (BIB-GSH) (N=56)	Weekly email contact with psycholo-gists or psychothe-rapists	INT-GSH = BIB-GSH		5,6,7
Leung et al [56]<br><br>China<br>Australia	ED (N=280) (SCOFF, EDE-Q5)	280/176 (62.9%)	Outpatient ED clinics, treatment units, university student newspaper, Internet websites	**Uncontrolled study**<br>Baseline,<br>1 month,<br>3 months (not reported),<br>6 months (not reported)	**“SMART EATING”**<br>6 components<br>Open end		Monthly email by a therapist	Improved		1,3,5,6,7
Pretorius et al [57]<br><br>United Kingdom	BN (N=61)<br>EDNOS (N=40)<br>(DSM-IV)	101 adoles-cents	ED clinics	**Uncontrolled study**<br>Baseline,<br>3 months (post),<br>3-month follow-up	**“Overcoming Bulimia”**<br>(adapted for adolescents)<br>8 modules<br>3 months		Weekly flexible email support from a clinician	Improved	On average 3 out of a possible 8 sessions were completed	1
Wagner et al [58]<br><br>Austria<br>Germany	BN or EDNOS (N=155) (DSM-IV)	155/126 (81.3%)<br>29 ado-lescents, 97 adults	Advertise-ments	**RCT**<br>Baseline,<br>4 months,<br>7 months,<br>18 months	**“SALUT”**<br>7 modules<br>4-7 months	Internet-based guided self-help (INT-GSH) (n=52 adults, n=18 adolescents)<br>Conventional guided self-help (BIB-GSH)	Weekly email contact with psycholo-gists or psychothe-rapists	Adults = adolescents		5,6,7

						(n=45 adults, n=11 adolescents)				
**Internet-based Therapist-delivered Treatment**										
Robinson & Serfaty [59]<br><br>United Kingdom	BN (N=18) BED (N=4) EDNOS (N=1) (BITE >10)	23/19 (82.6%)	University, email	**Uncontrolled study**<br>Baseline,<br>3 months (post)	**Email Therapy**<br>3 months		Email contact with a therapist twice a week	Improved		1,2,3,5,6,7,9
Robinson & Serfaty [60]<br><br>United Kingdom	BN (N=51) EDNOS (N=20) BED (N=26) (DSM-IV)	97	University, email	**RCT**<br>Baseline,<br>3 months (post)	**Email Therapy**<br>3 months	<u>Email bulimia therapy (eBT)</u> (N=36)<br><u>Self-directed writing (SDW)</u> (N=34)<br><u>Waiting list control (WLC)</u> (N=27)	Email contact with a therapist twice a week	eBT = SDW > WLC		2,3,5,7
Simpson et al [61]<br><br>United Kingdom	BN (N=5) AN (N=1) EDNOS (N=6) (SEDs)	12	ED service waiting list	**Uncontrolled study**<br>Baseline,<br>after therapy (post)	**Videoconferencing**<br>12-20 sessions CBT<br>6-8 sessions of nutritional education if necessary		CBT therapist	Improved		1,2,3,4,5,6,7,9
Mitchell et al [62]<br><br>USA	BN (N=71) EDNOS (N=57) (DSM-IV)	128/116 (90.6%)<br><u>F2F-CBT</u> 66/58 (87.9%)<br><u>TV-CBT</u> 62/58 (93.5%)	Mailings, advertise-ments	**RCT**<br>Baseline,<br>4 months (post),<br>3-month follow-up,<br>12-month follow-up	**Videoconferencing**<br>Manual-based cognitive behavioral therapy (CBT)<br>20 sessions<br>4 months	<u>Face-to-face CBT (F2F-CBT)</u> (N=66)<br><br><u>Telemedicine CBT (TV-CBT)</u> (N=62)	CBT (F2F or via telemedi-cine) by trained therapists	F2F-CBT = TV-CBT	<u>F2F-CBT</u> 40/66 (60.6%)<br><u>TV-CBT</u> 37/62 (59.7%)	4,5
**Internet-based Relapse Prevention**										

Fichter et al [63, 64]<br><br>Germany	AN (N=258) (DSM-IV)	258	Hospitals	**RCT**<br>Baseline,<br>9 months (post),<br>9-month follow-up	**“VIA”**<br>9 modules<br>9 months	Relapse prevention (RP) (N=128)<br>Treatment as usual (TAU) (N=130)	Email contact to a therapist	RP > TAU (completer)	51/128 (39.8%)	6,7,8
Mezei et al [65]<br><br>Hungary	BN or EDNOS (N=39) (DSM-IV)	39	Inpatient and outpatient treatment centers, online and paper advertise-ment	**Uncontrolled study**<br>Baseline,<br>4 months (post)	**“EDINA”**<br>4 months		Weekly group chat (90 min) with a therapist, voluntary individual chat session (30 min)	Unchanged		1,2,3,5,6,8, 9
**Body Image and Eating Disorder Prevention**										
Gollings & Paxton [66]<br><br>Australia	BSQ> 81.5 (N=40)	40	Advertise-ments on university campus and community notice-boards	**Pilot RCT**<br>Baseline,<br>2 months (post),<br>2-month follow-up	**“Set Your Body Free”**<br>Weekly 90-min group sessions<br>8 weeks	Face-to-face group (F2F) (N=19)<br>Internet group (N=21)	F2F and Internet group sessions led by a therapist	F2F = Internet group	F2F 15/19 (78.9%)<br>Internet group 18/21 (85.7%)	3,5,6,7,9
Paxton et al [67]<br><br>Australia	BSQ≥100 or BULIT-R>104 and BSQ =90-99 (N=116)	116	Advertise-ments on community notice-boards and referral services	**RCT**<br>Baseline,<br>8-9 weeks (post),<br>6-month follow-up	**“Set Your Body Free”**<br>Weekly 90-min group sessions<br>8 weeks	Face-to-face group (F2F) (N=42)<br>Internet group (N=37)<br>Delayed treatment control (DTC) group (N=37)	F2F and Internet group sessions led by a therapist	F2F > Internet group > DTC group	F2F 32/42 (76.2%)<br>Internet group 26/37 (70.3%)<br>DTC group 30/37 (81.1%)	3,5,6

Stice<br>et al [68, 69]<br><br>USA	Students with body dissatis-faction (N=107)	107	Participants from a local university invited via email messages and posters	**RCT**<br>Baseline,<br>4-6 weeks (post),<br>1-year follow-up,<br>2-year follow-up	**“eBody Project”**<br>6 modules<br>3 weeks<br>**“Body Project”**<br>Weekly 1-hour group sessions<br>4 weeks<br>**Educational video condition**<br>55-min documentary<br>**Educational brochure control condition**<br>2-page brochure	eBody Project (N=19)<br>Body Project (N=39)<br>Educational video condition (N=29)<br>Educational brochure condition (N=20)	eBody Project<br>No guidance<br>Body Project<br>Delivered by pairs of clinicians<br>Video/ brochure controls<br>No guidance	eBody Project = Body Project > Educational video condition = Educational brochure condition	eBody Project<br>17/19 (89.5%)<br>Body Project<br>31/39 (79.5%)	4,5
Serdar<br>et al [70]<br><br>USA	Weight and/or shape concerns (N=333)	333	Participant pool at a public university	**RCT**<br>Baseline,<br>session 3 (post)	**Dissonance-based ED prevention (DB)**<br>3 sessions for 1 hour	Face-to-face (F2F) DB (N=107)<br>Online DB (N=112)<br>Assessment only (N=114)	F2F DB and Online DB delivered by doctoral-level psychology graduate students	F2F DB = Online DB		2,3,4,5
Zabinski<br>et al [71]<br><br>USA	Weight Concerns Scale≥57 (N=60)	60	Public, west coast univer-sity	**RCT**<br>Baseline,<br>8 weeks (post),<br>10-week follow-up	**Synchronous Internet relay chat (IRC)**<br>Weekly 1-hour chat discussions<br>8 weeks	Internet relay chat (IRC) (N=30)<br>Waiting list control (WLC) (N=30)	IRC delivered by an advanced graduate student in clinical psychology	IRC > WLC		6,7,8
Ohlmer<br>et al [72]<br><br>Germany	Women at risk for AN (BMI, WCS, Restraint scale of	36	Lectures and seminars from different departments of 3 German universities	**Uncontrolled study**<br>Baseline,<br>10 weeks (post),<br>6-month follow-up	**“Student Bodies” for AN (SB-AN)**<br>Weekly sessions for 45-90 min<br>10 weeks		Individual weekly feedback by the moderator	Improved		1,4,5,8,9

	EDE-Q) (N=36)									
Heinicke et al [73] Australia	Self-identification as having body image or eating problems (N=83)	83/73 (88.0%) adolescents Intervention group 40/36 (90.0%) DTC group 43/37 (86.0%)	Flyers about the program to school counsellors and principals	**RCT** Baseline, 6 weeks (post)	**"My Body, My Life"** Weekly 90-min online sessions 6 weeks	Intervention group (N=40) Delayed treatment control (DTC) group (N=43)	Sessions conducted by a trained therapist	Intervention group > DTC group	Intervention group 30/40 (75.0%) DTC group 34/43 (79.1%)	3,6,8
**Eating Disorder Symptoms/Subthreshold Eating Disorders**										
Ruwaard et al [74] Netherlands	Bulimic symptoms (N=105) (EDE-Q, BAT)	105	Community	**RCT** Baseline, 20 weeks (post), 1-year follow-up	20 weeks	Online CBT (N=35) Bibliotherapy (Biblio.) (N=35) Waiting list (WL) (N=35)	25 scheduled therapist feedback moments during online CBT	Post Online CBT > Biblio. = WL Follow-up Online CBT = Biblio.	Online CBT 26/35 (74.3%) Biblio. 17/35 (48.6%)	3,6
Jacobi et al [75] Germany	Subthreshold ED (N=29) ED symptoms at lower level (N=97)	126	Universities and announcements (seminars, advertisements, email), fitness-studios,	**RCT** Baseline, 2 months (post), 6-month follow-up	**"Student Bodies+" (SB+)** 8 sessions 8 weeks	Intervention group (SB+) (N=64) Waiting list control (WLC) (N=62)	Individual weekly feedback by a group moderator	SB+ > WLC		3,6,7,8

			private practices of physicians, pharmacies							
**Motivation**										
Hötzel et al [76]<br><br>Germany	ED symp-toms (N=212) (SEED)	212	Media announce-ments (radio, newspaper, magazines, social networks, or websites)	**RCT**<br>Baseline,<br>6 weeks (post)	**“ESS-KIMO“**<br>Weekly online sessions for 45 min<br>6 weeks	<u>Intervention group (ESS-KIMO)</u><br>(N=103)<br><u>Waiting list control (WLC)</u><br>(N=109)	Asynchro-nous weekly feedback on website by therapist (individua-lized but standar-dized by predefined text elements)	ESS-KIMO > WLC (completer)		2,3,5,6,7,8
Leung et al [77]<br><br>China<br>Australia	ED (N=185) (SCOFF, EDE-Q5)	185/101 (54.6%)	Leaflets, posters, advertise-ments in outpatient ED clinics, ED treatment units, university student newspaper, Internet	**Uncontrolled study**<br>Baseline,<br>1 month,<br>3 months	**“SMART EATING”**<br>11 worksheets		Monthly emails by a therapist	Improved	36/185 (19.5%)	1,3,5,8
**Carers/Parents**										

Binford Hopf et al [78]<br><br>USA	Parents of AN patients (N=13)	13	Parents who were involved in FBT at the University of Chicago	**Uncontrolled study**<br>Baseline, before and after each chat session,<br>7 weeks (midstudy),<br>15 weeks (post)	**Internet-based Chat Support Groups**<br>15 weekly online chat sessions for 90 min		Licensed clinical psycholo-gist	Improved	9/13 (69.2%)	1,2,3,6,8,9
Grover et al [79]<br><br>United Kingdom	Carers of AN patients (N=27)	27	Clinical departments, volunteer database, advertise-ments	**Uncontrolled study**<br>Baseline,<br>9 weeks (post),<br>10-12-week follow-up	**Web-based systemic CBT intervention**<br>9 workbooks (encouraged 1 per week, but no formal time limit)		Flexible support (trained CBT practitio-ner), up to 20 min per week (email or phone)	Improved	22/27 (81.5%)	1,3,4,8,9
Grover et al [80]<br><br>United Kingdom	Carers of AN patients (N=64)	64/62 (96.9%)<br>OAO<br>34/33 (97.1%)<br>BEAT<br>30/29 (96.7%)	Online advertise-ments, carers' groups, clinical departments	**RCT**<br>Baseline,<br>4 months (post),<br>6-month follow-up	**"Overcoming anorexia online" (OAO)**<br>8 modules<br>4 months	OAO (N=34)<br>Control intervention (BEAT) (N=30)	A psycho-logist and a psychothe-rapist with CBT skills (up to 20 min per week, email or phone)	OAO > BEAT	OAO<br>17/34 (50.0%)<br>BEAT<br>5/30 (16.6%)	3,4
Hoyle et al [81]<br><br>Australia United Kingdom	Carers of AN patients (N=37)	37/33 (89.2%)<br>OAO<br>18/17 (94.4%)<br>OAO G<br>19/16 (84.2%)	Websites of consumer support associations, Facebook	**RCT**<br>Baseline,<br>7 weeks (post),<br>3-month follow-up	**"Overcoming anorexia online" (OAO)**<br>7 modules + 2 additional modules for carers<br>7 weeks	OAO without guidance (OAO-NoG) (N=18)<br>OAO with guidance (OAO-G) (N=19)	Email or telephone by a master level trainee psycholo-gist once a week	OAO-NoG = OAO-G		3,4,6,7,9

Bruning Brown et al [82]<br><br>USA	Parents of sophomore students (N=69)	69/58 (84.1%)<br>Intervention group<br>22/11 (50.0%)	Private, sectarian school	**RCT**<br>Baseline,<br>2 months (post),<br>3-month follow-up	**“Student Bodies” Parent Intervention**<br>4 weeks	Intervention group (N=22)<br>Controls (N=47)		Intervention group > Controls	8/22 (36.4%)	2,3,4,6,8
**Mobile/SMS Text Messaging**										
Shapiro et al [83]<br><br>USA	BN (N=31) (DSM-IV)	31/25 (80.6%)	From physician or from the university ED program, advertisements in the community	**Uncontrolled study**<br>Baseline,<br>12 weeks (post),<br>12-week follow-up	**Text Messaging**<br>Daily, 24 weeks<br>+ 12 Face-to-face (F2F) CBT sessions for 1,5 hours		F2F groups led by a clinical psychologist, automatic feedback messages	Improved	15/31 (48.4%)	1,3,5,8,9
Robinson et al [84]<br><br>United Kingdom	BN or EDNOS (N=34) (DSM-IV)	34/21 (61.8%)	Patients who had outpatient or day patient treatment at a specialist ED service	**Uncontrolled study**<br>Baseline,<br>6 months (post)	**SMS-based intervention**<br>Weekly<br>6 months			Unchanged	9/34 (26.5%)	1,2,3,4,5,8, 9
Bauer et al [85]<br><br>Germany	BN or EDNOS (N=165) (DSM-IV)	165/161 (97.6%)<br>SMS<br>82/78 (95.1%)<br>TAU<br>83/83 (100%)	Consecutive admissions to specialized inpatient care based on CBT	**RCT**<br>Admission,<br>discharge,<br>4 months (post),<br>4-month follow-up	**Aftercare SMS-based intervention**<br>Weekly symptom report via SMS<br>16 weeks	SMS-based maintenance intervention (SMS) (N=82)<br>Treatment as usual (TAU) (N=83)	Automatic, tailored feedback	Patients with outpatient treatment<br>SMS = TAU<br>Patients without outpatient treatment<br>SMS > TAU	SMS<br>61%	5,8
Cardi et al [86]<br><br>United Kingdom	AN (N=18)<br>BN (N=13) (DSM-IV)	31	Institute of Psychiatry ED unit’s volunteer database, advertisements and	**Uncontrolled study**<br>Baseline,<br>3 weeks (post)	Mp4 player or iPod with 10 video clips (vodcasts) between 3 to 20 min each, workbook and daily monitoring forms<br>3 weeks		Three 30-45 min sessions provided by a PhD psychologist	Improved	29/31 (93.5%)	1,2,3,5,8,9

			posters in local ED services							

[a] AN = Anorexia nervosa, BAT = Body Attitude Test, BEAT = Beating Eating Disorders, BED = Binge eating disorder, BIB-GSH = Conventional guided bibliotherapy, Biblio. = bibliotherapy, BITE = Bulimic Investigatory Test-Edinburgh, BMI = Body-Mass-Index, BN = Bulimia nervosa, BSQ = Body Shape Questionnaire, BULIT-R = Bulimia Test-Revised, CBT = Cognitive behavioral therapy, CD-ROM = Compact Disc Read Only Memory, DB = Dissonance-based, DSM = Diagnostic and Statistical Manual of Mental Disorders, DTC = Delayed treatment control, eBT = Email bulimia therapy, ED = Eating Disorder, EDE-Q = Eating Disorder Examination Questionnaire, EDNOS = Eating disorders not otherwise specified, FBT = Family-based treatment, F2F = Face-to-face, IBT = Internet-based therapy, iCBT = Internet-based cognitive behavioral therapy, INT-GSH = Internet-based guided self-help, IRC = Internet relay chat, MG = Minimal guidance, OAO = Overcoming Anorexia Online, OAO G = Overcoming Anorexia Online with Guidance, RCT = Randomized controlled trial, RP = Relapse prevention, SB-AN = Student Bodies for AN, SB+ = Student Bodies adapted for subthreshold ED, SCOFF = Sick Control One Fat Food, SDW = Self-directed writing, SEDs = Survey for Eating Disorders, SEED = Short Evaluation of Eating Disorders, SMS = Short Message Service, TAU = Treatment as usual, TG = Therapist guidance, TV-CBT = Telemedicine cognitive behavioral therapy, WCS = Weight Concerns Scale, WL = Waiting list, WLC = Waiting list control.

Codes for weaknesses following Newman and et al [121]: 1. Uncontrolled, 2. No follow-up, 3. Used only self-reports as outcome measure, 4. No information about additional psychological therapy, 5. No information about additional pharmacological treatment, 6. No adherence or quality checks on treatment or no information about it, 7. No information on therapist training, 8. Only used a waiting list or no treatment comparison, 9. Small sample (N < 50).
