# Supplementary material for: The Potential of Technology-Based Psychological Interventions for Anorexia and Bulimia Nervosa: A Systematic Review and Recommendations for Future Research
Source: J Med Internet Res. 2015 Mar 31;17(3):e85. doi: 10.2196/jmir.3554 (PMC4397416; doi:10.2196/jmir.3554)
Supplement: Supplementary file 2 [file jmir_v17i3e85_app2.pdf]

Within- and between-group effect sizes and confidence intervals for included studies (posttreatment and follow-up) [a]

Study	Symptoms	Within-group effect size<br>Baseline – Posttreatment<br>[lower CI; upper CI]	Within-group effect size<br>Baseline – Follow-up 1<br>Baseline – Follow-up 2<br>[lower CI; upper CI]	Between-group effect size<br>Posttreatment<br>[lower CI; upper CI]	Between-group effect size<br>Follow-up 1<br>Follow-up 2<br>[lower CI; upper CI]
**Computer- and Internet-based Unguided Self-help**					
Bara-Carril et al [42]	Binging		**0.75 [0.23; 1.26]**		
	Vomiting		**0.63 [0.12; 1.14]**		
	ED psychopathology				
Schmidt et al [43]	Binging				
	Vomiting				
	ED psychopathology (EDE Global)	CD-ROM: 0.28 [-0.16; 0.71]<br>WL: 0.27 [-0.15; 0.69]	CD-ROM: 0.35 [-0.10; 0.81]<br>WL: **0.51 [0.04; 0.97]**	0.09 [-0.37; 0.54]	0.22 [-0.30; 0.73]
Johnston et al [44]	Binging				
	Vomiting				
	ED psychopathology (BITE Total)	Therapy: 0.17 [-0.27; 0.61]<br>Control: 0.25 [-0.21; 0.71]	Therapy: 0.23 [-0.21; 0.67]<br>Control: 0.17 [-0.28; 0.63]	0.16 [-0.29; 0.61]	0.03 [-0.42; 0.48]
**Computer- and Internet-based Guided Self-help**					
Sánchez-Ortiz et al [48]	Binging	iCBT: **0.79 [0.31; 1.26]**<br>WL: 0.31 [-0.17; 0.79]	iCBT: **1.05 [0.54; 1.57]**<br>WL: **0.73 [0.20; 1.27]**	-0.40 [-0.88; 0.09]	**-0.59 [-1.15; -0.03]**
	Vomiting	iCBT: **0.49 [0.02; 0.95]**<br>WL: 0.22 [-0.26; 0.70]	iCBT: **0.75 [0.25; 1.25]**<br>WL: **0.56 [0.03; 1.08]**	-0.33 [-0.82; 0.15]	-0.40 [-0.96; 0.15]
	ED psychopathology (EDE Global)	iCBT: **1.27 [0.77; 1.77]**<br>WL: 0.47 [-0.01; 0.95]	iCBT: **1.73 [1.16; 2.29]**<br>WL: **0.90 [0.36; 1.44]**	**-1.23 [-1.76; -0.71]**	**-0.97 [-1.54; -0.39]**
Ljottson et al [49]	Binging	Treatment: **1.42 [0.88; 1.96]**<br>Control: 0.17 [-0.65; 0.31]		**-0.67 [-1.17; -0.18]**	
	Vomiting	**Purging**<br>Treatment: 0.60 [-0.11; 1.09]<br>Control: -0.22 [-0.70; 0.26]		**Purging**<br>**-2.06 [-2.66; -1.47]**	
	ED psychopathology (EDE-Q Global)	Treatment: **1.04 [0.52; 1.55]**<br>Control: 0.10 [-0.38; 0.57]		**-1.13 [-1.65; -0.61]**	
Nevonen et al [52]	Binging		0.30 [-0.20; 0.80]		
	Vomiting		0.46 [-0.04; 0.96]		
	ED psychopathology				
Carrard et al [54]	Binging	**0.64 [0.33; 0.95]**			
	Vomiting	**0.82 [0.50; 1.14]**			
	ED psychopathology				

Wagner et al [55]	Binging	INT-GSH: 0.24 [-0.09; 0.57]<br>BIB-GSH: 0.32 [-0.07; 0.70]	INT-GSH: 0.33 [-0.01; 0.66]<br>BIB-GSH: 0.31 [-0.08; 0.69]<br><br>INT-GSH: **0.50 [0.16; 0.83]**<br>BIB-GSH: **0.43 [0.04; 0.82]**	0.03 [-0.33; 0.39]	-0.03 [-0.39; 0.33]<br><br>-0.13 [-0.49; 0.23]
	Vomiting	INT-GSH: **0.35 [0.02; 0.69]**<br>BIB-GSH: 0.35 [-0.03; 0.74]	INT-GSH: **0.39 [0.06; 0.73]**<br>BIB-GSH: **0.39 [0.00; 0.78]**<br><br>INT-GSH: **0.56 [0.23; 0.90]**<br>BIB-GSH: **0.54 [0.15; 0.93]**	0.14 [-0.22; 0.50]	0.14 [-0.22; 0.50]<br><br>-0.04 [-0.40; 0.32]
	ED psychopathology (EDI-2 Total)	INT-GSH: **0.50 [0.17; 0.84]**<br>BIB-GSH: **0.49 [0.10; 0.87]**	INT-GSH: **0.72 [0.38; 1.06]**<br>BIB-GSH: **0.61 [0.22; 1.00]**<br><br>INT-GSH: **0.64 [0.30; 0.98]**<br>BIB-GSH: **0.57 [0.18; 0.96]**	-0.03 [-0.38; 0.33]	-0.13 [-0.48; 0.23]<br><br>-0.06 [-0.42; 0.29]
Leung et al [56]	Binging	0.28 [-0.07; 0.63]			
	Vomiting	-0.01 [-0.35; 0.34]			
	ED psychopathology (EDE-Q Global)	**0.64 [0.29; 0.99]**			
Pretorius et al [57]	Binging	**4.41 [3.82; 5.01]**	**4.39 [3.82; 4.96]**		
	Vomiting	**3.54 [3.03; 4.06]**	**3.96 [3.43; 4.49]**		
	ED psychopathology (EDE Global)	**7.05 [6.19; 7.91]**	**5.46 [4.79; 6.13]**		
Wagner et al [58]	Binging	Adults: 0.37 [-0.05; 0.79]<br>Adolescents: 0.31 [-0.39; 1.01]	Adults: **0.66 [0.23; 1.09]**<br>Adolescents: 0.26 [-0.43; 0.96]<br><br>Adults: **0.55 [0.12; 0.97**]<br>Adolescents: 0.63 [-0.08; 1.33]	-0.49 [-1.07; 0.09]	**-0.60[-1.18; -0.02]**<br>-0.25 [-0.82; 0.32]
	Vomiting	Adults: 0.30 [-0.11; 0.72]<br>Adolescents: 0.55 [-0.16; 1.26]	Adults: **0.44 [0.02; 0.85]**<br>Adolescents: 0.51 [-0.19; 1.21]	-0.22 [-0.79; 0.35]	-0.38 [-0.95; 0.20]<br>-0.26 [-0.83; 0.31]
	ED psychopathology (EDI-2 Total)	Adults: **0.75 [0.33; 1.17]**<br>Adolescents: 0.61 [-0.15; 1.36]	Adults: **1.37 [0.92; 1.82]**<br>Adolescents: **0.77 [0.00; 1.53]**<br><br>Adults: **1.02 [0.59; 1.44]**<br>Adolescents: **0.79 [0.02; 1.56]**	0.01 [-0.58; 0.61]	-0.28 [-0.88; 0.32]<br>-0.10 [-0.69; 0.50]
**Internet-based Therapist-delivered Treatment**					
Robinson & Serfaty [59]	Binging				
	Vomiting				
	ED psychopathology	**BITE severity**			

	(BITE)	0.41 [-0.17; 1.00]<br><br>**BITE symptoms**<br>0.56 [-0.03; 1.15]			
Mitchell et al [62]	Binging	TV-CBT: **0.62 [0.22; 1.03]**<br>F2F-CBT: **0.80 [0.39; 1.21]**	TV-CBT: **0.60 [0.19; 1.02]**<br>F2F-CBT: **0.73 [0.30; 1.15]**<br><br>TV-CBT: 0.31 [-0.15; 0.76]<br>F2F-CBT: **0.62 [0.15; 1.09]**	0.21 [-0.23; 0.65]	0.12 [-0.35; 0.58]<br><br>0.28 [-0.27; 0.82]
	Vomiting	TV-CBT: **0.87 [0.46; 1.28]**<br>F2F-CBT: **1.07 [0.65; 1.49]**	TV-CBT: **0.88 [0.46; 1.31]**<br>F2F-CBT: **0.92 [0.49; 1.35]**<br><br>TV-CBT: **0.50 [0.05; 0.96]**<br>F2F-CBT: **0.90 [0.42; 1.37]**	0.34 [-0.10; 0.78]	0.12 [-0.35; 0.58]<br><br>0.41 [-0.14; 0.96]
	ED psychopathology				
**Internet-based Relapse Prevention**					
Fichter et al [63, 64]	Binging				
	Vomiting				
	ED psychopathology (EDI-2 Total, BMI)	**EDI-2 Total**<br>RP: **-0.34 [-0.61; -0.07]**<br>TAU: **-0.57 [-0.84; -0.30]**<br><br>**BMI**<br>RP: -0.18 [-0.48; 0.11]<br>TAU: 0.06 [-0.20; 0.31]	**BMI**<br>RP: **-0.44 [-0.74; -0.14]**<br>TAU: **-0.30 [-0.55; -0.04]**	**EDI-2 Total**<br>-0.27 [-0.53; 0.00]<br><br>**BMI**<br>0.21 [-0.04; 0.47]	**BMI**<br>0.15 [-0.12; 0.43]
Mezei et al [65]	Binging	0.06 [-0.46; 0.58]			
	Vomiting	0.12 [-0.41; 0.64]			
	ED psychopathology (EDE-Q Global)	0.28 [-0.25; 0.81]			
**Body Image and Eating Disorder Prevention**					
Gollings & Paxton [66]	Body dissatisfaction (BSQ)	Int.: **0.97 [0.31; 1.62]**<br>F2F: **0.73 [0.07; 1.39]**	Int.: **0.96 [0.31; 1.62]**<br>F2F: **0.92 [0.25; 1.59]**	-0.27 [-0.90; 0.36]	-0.11 [-0.74; 0.52]
Paxton et al [67]	Body dissatisfaction (BSQ)	Int.: **0.58 [0.12; 1.05]**<br>F2F: **1.19 [0.72; 1.65]**<br>Del.: 0.16 [-0.29; 0.62]		Int. vs. F2F.:<br>0.32 [-0.13; 0.76]<br><br>Int. vs. Del.:<br>**-0.53 [-1.00; -0.07]**	

Stice<br>et al [68, 69]	Thin-ideal internalization (IBSS-R)	eBody: **0.69 [0.03; 1.34]**<br>Body: **0.75 [0.29; 1.21]**<br>Brochure: 0.08 [-0.54; 0.70]<br>Video: 0.21 [-0.31; 0.73]	eBody: 0.24 [-0.40; 0.88]<br>Body: **0.89 [0.42; 1.35]**<br>Brochure: 0.42 [-0.21; 1.04]<br>Video: 0.37 [-0.15; 0.89]<br><br>eBody: 0.33 [-0.31; 0.97]<br>Body: **0.78 [0.32; 1.24]**<br>Brochure: **0.81 [0.16; 1.45]**<br>Video: **0.57 [0.04; 1.09]**	eBody vs. Body: -0.18 [-0.73; 0.37]<br>eBody vs. Brochure: **-0.84 [-1.49; -0.18]**<br>eBody vs. Video: -0.55 [-1.14; 0.04]	eBody vs. Body: 0.34 [-0.21; 0.89]<br>eBody vs. Brochure: -0.14 [-0.77; 0.49]<br>eBody vs. Video: 0.03 [-0.55; 0.61]<br><br>eBody vs. Body: 0.23 [-0.32; 0.78]<br>eBody vs. Brochure: 0.05 [-0.58; 0.68]<br>eBody vs. Video: 0.10 [-0.48; 0.68]
Serdar<br>et al [70]	Thin-ideal internalization (IBSS-R)	Online: 0.18 [-0.09; 0.44]<br>F2F: 0.24 [-0.02; 0.51]<br>Ass.: 0.09 [-0.17; 0.35]		Online vs. F2F: 0.05 [-0.21; 0.32]<br>Online vs. Ass.: -0.23 [-0.49; 0.03]	
Zabinski<br>et al [71]	ED psychopathology (EDE-Q Global)	Int.: **0.75 [0.23; 1.27]**<br>Control: 0.03 [-0.48; 0.53]	Int.: **0.58 [0.06; 1.10]**<br>Control: -0.04 [-0.55; 0.46]	-0.25 [-0.75; 0.26]	-0.36 [-0.87; 0.15]
Ohlmer<br>et al [72]	ED psychopathology (EDE-Q Global)	**0.82 [0.34; 1.30]**	**0.74 [0.26; 1.21]**		
Heinicke<br>et al [73]	Body dissatisfaction (BSQ)	Int.: **0.59 [0.12; 1.07]**<br>Del.: -0.04 [-0.50; 0.42]		**-0.70 [-1.18; -0.23]**	
**Eating Disorder Symptoms/Subthreshold Eating Disorders**					
Ruwaard<br>et al [74]	Binging	Online: **1.05 [0.55; 1.55]**<br>Biblio.: 0.16 [-0.31; 0.63]<br>WL: 0.31 [-0.17 ; 0.78]	Online: **1.00 [0.50; 1.50]**<br>Biblio.: 0.28 [-0.20; 0.75]	Online vs. Biblio.: **-0.70 [-1.18; -0.21]**<br>Online vs. WL: -0.43 [-0.90; 0.05]	Online vs. Biblio.: **-0.43 [-0.82; -0.04]**
	Vomiting				
	ED psychopathology (EDE-Q Global)	Online: **1.18 [0.67; 1.68]**<br>Biblio.: **0.54 [0.07; 1.02]**<br>WL: 0.34 [-0.13; 0.82]	Online: **1.10 [0.60; 1.60]**<br>Biblio.: **1.03 [0.54; 1.53]**	Online vs. Biblio.: -0.37 [-0.84; 0.10]<br>Online vs. WL:	Online vs. Biblio.: 0.14 [-0.33; 0.61]

				**-0.50 [-0.97; -0.02]**	
Jacobi et al [75]	Binging	SB+: **0.77 [0.37; 1.17]**<br>Control: 0.00 [-0.38; 0.38]	SB+: **1.00 [0.59; 1.41]**<br>Control: 0.20 [-0.19; 0.58]	-0.29 [-0.67; 0.10]	**-0.43 [-0.82; -0.04]**
	Vomiting	SB+: 0.28 [-0.11; 0.67]<br>Control: -0.13 [-0.52; 0.25]	SB+: 0.14 [-0.25; 0.53]<br>Control: -0.31 [-0.69; 0.08]	-0.21 [-0.60; 0.18]	-0.33 [-0.72; 0.06]
	ED psychopathology (EDE-Q Global)	SB+: **0.62 [0.23; 1.02]**<br>Control: 0.30 [-0.09; 0.69]	SB+: **0.99 [0.58; 1.41]**<br>Control: 0.20 [-0.19; 0.59]	-0.10 [-0.48; 0.29]	**-0.50 [-0.89; -0.11]**
**Motivation**					
Hötzel et al [76]	Binging				
	Vomiting	Intervention: 0.06 [-0.21; 0.33]<br>Control: 0.07 [-0.20; 0.33]		-0.22 [-0.49; 0.05]	
	Motivation (SOCQ-ED Item 6)	Intervention: **-0.37 [-0.65; -0.10]**<br>Control: 0.02 [-0.24; 0.29]		0.13 [-0.14; 0.40]	
Leung et al [77]	Binging	0.37 [-0.04; 0.78]	0.11 [-0.30; 0.52]		
	Vomiting	-0.03 [-0.44; 0.38]	0.17 [-0.24; 0.58]		
	Motivation	**Giving up binging**<br>-0.06 [-0.48; 0.37]<br><br>**Giving up vomiting**<br>0.02 [-0.40; 0.45]	**Giving up binging**<br>-0.28 [-0.71; 0.14]<br><br>**Giving up vomiting**<br>-0.01 [-0.43; 0.41]		
**Carers/Parents**					
Binford Hopf et al [78]	ED symptom impact (EDSIS Total)	**1.18 [0.23; 2.13]**			
Grover et al [79]	Anxiety and depression (HADS Total)	**0.70 [0.11; 1.28]**	**0.78 [0.16; 1.40]**		
Hoyle et al [81]	High-expressed emotion (LEE)	OAO-NoG: 0.16 [-0.50; 0.81]<br>OAO-G: 0.34 [-0.32; 1.00]	OAO-NoG: -0.08 [-0.73; 0.58]<br>OAO-G: 0.49 [-0.17; 1.16]	0.50 [-0.16; 1.16]	**0.94 [0.25; 1.63]**
Bruning Brown et al [82]	Parental attitudes and criticism (PACS)	**Critical to others**<br>Intervention: **0.65 [0.04; 1.26]**<br>Control: 0.00 [-0.40; 0.40]<br><br>**Healthy outlook**<br>Intervention: **-0.74 [-1.35; -0.13]**<br>Control: -0.16 [-0.56; 0.25]		**Critical to others**<br>-0.27 [-0.63; 0.08]<br><br>**Healthy outlook**<br>0.29 [-0.35; 0.93]	

**Mobile/SMS Text Messaging**			
Shapiro et al [83]	Binging	**0.90 [0.15; 1.66]**	
	Vomiting	**Purging**<br>0.72 [-0.02; 1.45]	**Purging**<br>0.70 [-0.05; 1.45]
	ED psychopathology (EDI-2 Total)	**1.26 [0.48; 2.05]**	**1.76 [0.90; 2.61]**
Robinson et al [84]	Binging	-0.20 [-0.84; 0.44]	
	Vomiting	0.16 [-0.48; 0.80]	
	ED psychopathology		
Bauer et al [85]	Binging		
	Vomiting		
	ED psychopathology		
Cardi et al [86]	Binging	0.13 [-0.38; 0.65]	
	Vomiting	0.44 [-0.08; 0.96]	
	ED psychopathology (EDE-Q Global)	0.43 [-0.09; 0.95]	

[a] Ass. = Assessment only, BIB-GSH = Conventional guided bibliotherapy, Biblio. = Bibliotherapy, BITE = Bulimic Investigatory Test-Edinburgh, BMI = Body-Mass-Index, BSQ = Body Shape Questionnaire, CBT = Cognitive behavioral therapy, CD-ROM = Compact Disc Read Only Memory, CI = Confidence Interval, Del. = Delayed treatment group, ED = Eating Disorder, EDE = Eating Disorder Examination, EDE-Q = Eating Disorder Examination Questionnaire, EDI-2 = Eating Disorder Inventory-2, EDSIS = Eating Disorder Symptom Impact Scale, F2F = Face-to-face, HADS = Hospital Anxiety and Depression Scale, IBSS-R = Ideal-Body Stereotype Scale-Revised, iCBT = Internet-based cognitive behavioral therapy, Int. = Intervention group, INT-GSH = Internet-based guided self-help, LEE = Level of Expressed Emotion Scale, OAO-G = Overcoming Anorexia Online with Guidance, OAO-NoG = Overcoming Anorexia Online No Guidance, PACS = Parental Attitudes and Criticism Scale, RP = Relapse Prevention, SB+ = Student Bodies adapted for subthreshold EDs, SOCQ-ED = Stages of Change Questionnaire for Eating Disorders, TAU = Treatment as usual, TV-CBT = Telemedicine cognitive behavioral therapy, WL = Waiting list, Boldface data show CIs not covering zero.
