# Supplementary material for: The Potential of Technology-Based Psychological Interventions for Anorexia and Bulimia Nervosa: A Systematic Review and Recommendations for Future Research
Source: J Med Internet Res. 2015 Mar 31;17(3):e85. doi: 10.2196/jmir.3554 (PMC4397416; doi:10.2196/jmir.3554)
Supplement: Supplementary file 3 [file jmir_v17i3e85_app3.pdf]

Definition of abstinence in studies included in the review as well as abstinence rates and results of significance test wherever data was available [a]

Study	Definition of abstinence	Abstinence Rates		
		Posttreatment	Follow-up 1	Follow-up 2
**Computer- and Internet-based Unguided Self-help**				
Bara-Carril et al [42]	Not at all		**Binging:** 12.8%<br>**Vomiting:** 46.2%	
Schmidt et al [43]	Being free of binging, vomiting and laxative misuse over the previous 28 days	CD-ROM: 12.0%<br>WL: 10.0%	CD-ROM: 13.0%<br>WL: 20.0%	
**Computer- and Internet-based Guided Self-help**				
Huon [45]	Symptom free	Contact cured: 23.3%<br>Contact improved: 16.6%<br>Program only: 16.6%<br>Untreated: 0.0%	Contact cured: 23.3%<br>Contact improved: 20.0%<br>Program only: 16.6%<br>Untreated: 6.6%	Contact cured: 46.6%<br>Contact improved: 33.3%<br>Program only: 16.6%<br>Untreated: 6.6%
Sánchez-Ortiz et al [48]	No objective binges, episodes of vomiting and laxative use in the past month	iCBT: 25.8%<br>WL: 13.9%	iCBT: 39.1%<br>WL: 20.7%	
Ljotsson et al [49]	Patients with no episodes of objective binge eating and purging during the last 28 days	Treatment: 37.0%<br>Control: 15.0%	Treatment: 44.0%	
Carrard et al [50]	Abstainers no longer suffered from binges or episodes of vomiting	17.2%	17.2%	
Fernández-Aranda et al [53]	Abstinence from binging and purging for a minimum of at least two consecutive weeks	**Binging:** (p<.05)<br>IBT: 32.3%<br>WL: 3.2%<br>**Vomiting:** (p<.05)<br>IBT: 32.3%<br>WL: 0.0%<br>**Binging+vomiting:** (p<.05)<br>IBT: 22.6%<br>WL: 0.0%		
Carrard et al [54]	No symptoms anymore	23.0%		
Pretorius et al [57]	Free of binging, vomiting, and laxative use	6.0%	12.0%	
Wagner	Abstinent from binge eating and	Adolescents: 44.4% (n.s.)	Adolescents: 55.0% (n.s.)	

et al [58]	compensatory behavior or no fulfillment of DSM-IV criteria	Adults: 38.7%	Adults: 62.5%	
**Internet-based Therapist-delivered Treatment**				
Robinson & Serfaty [60]	Absence of an eating disorders diagnosis	eBT: 22.2%<br>SDW: 14.7% (vs. eBT n.s.)<br>WL: 0.0% (vs. eBT p<.05)		
Simpson et al [61]	Symptom free	66.7%		
Mitchell et al [62]	None of these behaviors (binge eating, purging: vomiting, laxative abuse, diuretic abuse) in the previous 28 days	**Binging:** (n.s.)<br>F2F-CBT: 50.0%<br>TV-CBT: 50.0%<br>**Vomiting:** (n.s.)<br>F2F-CBT: 36.4%<br>TV-CBT: 30.6%<br>**Binging+purging:** (n.s.)<br>F2F-CBT: 28.8%<br>TV-CBT: 27.4%	**Binging:** (n.s.)<br>F2F-CBT: 50.8%<br>TV-CBT: 48.4%<br>**Vomiting:** (n.s.)<br>F2F-CBT: 33.3%<br>TV-CBT: 22.6%<br>**Binging+purging:** (n.s.)<br>F2F-CBT: 25.8%<br>TV-CBT: 21.0%	**Binging:** (n.s.)<br>F2F-CBT: 53.3%<br>TV-CBT: 46.8%<br>**Vomiting:** (n.s.)<br>F2F-CBT: 34.8%<br>TV-CBT: 25.8%<br>**Binging+purging:** (n.s.)<br>F2F-CBT: 28.8%<br>TV-CBT: 22.6%
**Eating Disorder Symptoms/Subthreshold Eating Disorders**				
Ruwaard et al [74]	Abstinence (ie, a frequency of 0)	**Binging:** (n.s.)<br>Online: 37.0%<br>Biblio.: 14.0%<br>WL: 14.0%<br>**Purging:** (p<.05)<br>Online: 39.0%<br>Biblio.: 8.0%<br>WL: 8.0%	**Binging:** (n.s.)<br>Online: 40.0%<br>Biblio.: 34.0%<br>**Purging:** (n.s.)<br>Online: 39.0%<br>Biblio.: 30.0%	
Jacobi et al [75]	Abstinent from all symptoms of disordered eating (restrictive eating, binge eating and any compensatory behaviors)	SB+: 45.1%<br>Control: 19.2%	SB+: 45.1% (p<.05)<br>Control: 26.9%	
**Mobile/SMS Text Messaging**				
Robinson et al [84]	Binge and vomit free over the previous month	29.4%		
Bauer et al [85]	Absence of binge eating and compensatory behaviors for a minimum of 4 weeks	SMS: 37.8% (p<.05)<br>TAU: 18.1%		

[a] Biblio. = Bibliotherapy, CD-ROM = Compact Disc Read Only Memory, DSM = Diagnostic and Statistical Manual of Mental Disorders, eBT = Email bulimia therapy, F2F-CBT = Face-to-face cognitive behavioral therapy, IBT = Internet-based therapy, iCBT = Internet-based cognitive behavioral therapy, n.s. = not statistically significant, SB+ = Student Bodies

adapted for subthreshold eating disorders, SDW = Self-directed writing, SMS = Short Message Service, TAU = Treatment as usual, TV-CBT = Telemedicine cognitive behavioral therapy, WL = Waiting list.
